# Secretory mitophagy: an extracellular vesicle-mediated adaptive mechanism for cancer cell survival under oxidative stress

**DOI:** 10.3389/fcell.2024.1490902

**Published:** 2025-01-30

**Authors:** Purva V. Gade, Angela Victoria Rojas Rivera, Layla Hasanzadah, Sofie Strompf, Thomas Raymond Philipson, Matthew Gadziala, Atharva Tyagi, Arnav Bandam, Rithvik Gabbireddy, Fatah Kashanchi, Amanda Haymond, Lance A. Liotta, Marissa A. Howard

**Affiliations:** ^1^ Center for Applied Proteomics and Molecular Medicine, George Mason University, Manassas, VA, United States; ^2^ Laboratory of Molecular Virology, School of Systems Biology, George Mason University, Manassas, VA, United States

**Keywords:** mitophagy, cancer progression, extracellular vesicles, PINK1, oxidative stress, cell survival

## Abstract

Mitophagy is a critically important survival mechanism in which toxic, aged, or defective mitochondria are segregated into mitophagosomes, which shuttle the damaged mitochondrial segments to the lysosome and proteasome for destruction. Cancer cells rely on mitophagy under conditions of high oxidative stress or increased energy demand. Oxidative stress can generate a large volume of damaged mitochondria, overwhelming lysosomal removal. Accumulated damaged mitochondria are toxic and their proper removal is crucial for maintaining mitochondrial health. We propose a new cancer cell mechanism for survival that is activated when the demand for segregating and eliminating damaged mitochondria exceeds the capacity of the lysosome or proteasome. Specifically, we show that tumor cells subjected to oxidative stress by carbonyl cyanide-3-chlorophenylhdrazone (CCCP) eliminate damaged mitochondria segments by bypassing the lysosome to export them outside the cell via extracellular vesicles (EVs), a process termed “secretory mitophagy”. PINK1, the initiator of mitophagy, remains associated with the damaged mitochondria that exported in EVs. Using several types of cancer cells, we show that tumor cells treated with CCCP can be induced to switch over to secretory mitophagy by treatment with Bafilomycin A1, which blocks the fusion of mitophagosomes with lysosomes. Under these conditions, an increased number of PINK1 + EVs are exported. This is associated with greater cell survival by a given CCCP dose, enhanced mitochondrial ATP production, and reduced mitochondrial oxidative damage (membrane depolarization). Our data supports the hypothesis that secretory mitophagy is a previously unexplored process by which cancer cells adapt to survive therapeutic or hypoxic stress. Ultimately, our findings may inform new prevention strategies targeting pre-malignant lesions and therapeutic approaches designed to sensitize tumor cells to oxidative stress-inducing therapies.

## 1 Introduction

Cancer cells reprogram their metabolism to meet energy demands, produce necessary biomolecules, and maintain cellular redox balance, enhancing their survival, growth, and resistance to therapies (Schiliro and Firestein, 2021). Mitophagy, the specific identification, sequestration, and degradation of mitochondria plays a crucial role in maintaining cellular health by removing aged or damaged mitochondria, thus preserving the integrity and functionality of the mitochondrial pool (Ashrafi and Schwarz, 2013; Rodger et al., 2018; Choubey et al., 2021; Lemasters, 2014; Hong et al., 2022). Beyond clearing dysfunctional mitochondria, mitophagy also reduces mitochondrial mass under specific stresses, such as hypoxia and nutrient deprivation (Vara-Perez et al., 2019; Doblado et al., 2021). This adaptive response prevents the generation of toxic reactive oxygen species (ROS) and conserves essential nutrients required for cell proliferation (Zorov et al., 2014). Consequently, mitophagy is vital for survival during energetic stress. Proper regulation of mitochondrial turnover and biogenesis is pivotal in cancer progression (Chourasia et al., 2015; Macleod, 2020; Song et al., 2022; Denisenko et al., 2021). Therefore, understanding the mechanisms governing mitophagy and its regulation is crucial for elucidating how cells maintain mitochondrial functioning and adapt to stress.

PTEN-induced kinase 1 (PINK1) is a protein kinase sensitive to damaged mitochondria that triggers mitophagy. Under normal conditions, mitochondrial membrane proteases continuously cleave PINK1, and the cleaved protein is degraded by the proteasome. As a result, in healthy mitochondria, only minimal levels of uncleaved (full-length, 63 kDa) PINK1 are present in the cytosol. During canonical mitophagy, uncleaved PINK1 associates with the surface of the depolarized mitochondria (Deas et al., 2011; Pollock et al., 2021; Gonçalves and Morais, 2021; Becker et al., 2012). On the damaged mitochondrial surface, PINK1 dimerizes and phosphorylates several mitophagy-related proteins, targeting the damaged segment for fission and encapsulation within an autophagosome (Pryde et al., 2016; McLelland et al., 2014). In this fashion, damaged mitochondria are identified by PINK1 localization and dimerization to trigger subsequent mitophagosome shuttling to the lysosome for destruction.

Extracellular vesicles (EVs) are lipid-encapsulated dense molecular packages implicated in tumor cell survival (Chang et al., 2021). Recently, we discovered a novel EV-mediated export pathway termed, “secretory mitophagy”. Secretory mitophagy involves the extracellular export of defective mitochondria bypassing lysosomal or proteasomal degradation. The exported secretory mitophagy EV particles contain uncleaved PINK1 along with a full set of downstream mitophagy machinery and mitochondrial proteins (Howard et al., 2022). We hypothesize that, in response to oxidative stress, tumor cells export damaged mitochondrial segments via EVs to circumvent conventional canonical mitophagy.

We developed an *in vitro* model to test the hypothesis that tumor cells can use secretory mitophagy to enhance cell survival and maintain critical mitochondrial functions under oxidative stress. We chose this model because it allows us to mimic the stress conditions tumor cells face while resisting the cell death, enabling us to investigate the adaptive mechanisms employed. PINK1 + EVs serve as evidence for this process (Howard et al., 2022). Our findings reveal that enhanced export of PINK1 + EVs under oxidative stress improves cell survival, boosts ATP production, and reduces mitochondrial depolarization, while maintaining low cytosolic PINK1 levels. This data supports the hypothesis that PINK1 EV-mediated secretory mitophagy enables tumor survival under energetic stress.

## 2 Materials and methods

### 2.1 Cell culture

All cell lines were obtained from the American Type Culture Collection (ATCC, Manassas, VA, United States). The 4T1 murine mammary cancer cell line (ATCC^®^ CRL-2539™) was cultured in RPMI 1640 medium (Gibco™, Cat. #11875093) supplemented with 10% heat-inactivated, exosome-free fetal bovine serum (FBS, Gibco™, Cat. #A5256701), 100 U/mL penicillin-G, and 100 μg/mL streptomycin (Sigma-Aldrich, Cat. #P4333). The RAW 264.7 murine macrophage cells (ATCC^®^ TIB-71™), PANC1 human pancreatic cancer cells (ATCC^®^ CRL-1469™), and IOMM-Lee human meningioma cells (ATCC^®^ CRL-3370™) were maintained in Dulbecco’s Modified Eagle Medium (DMEM, Gibco™) supplemented with 10% heat-inactivated, exosome-free FBS and Penicillin-Streptomycin (Sigma-Aldrich, Cat. #P4333). All cultures were incubated at 37°C in a humidified atmosphere containing 5% CO₂. Cells were cultured as monolayers and maintained for ≤10 passages. When confluent, cells were harvested using Trypsin-EDTA (0.25%, phenol red; Gibco™, Cat. #25200072) by incubating at 37°C for 3 min and were subsequently replated for experimental procedures. Cell density was assessed using a QuadCount™ automated cell counter (Accuris Instruments).

### 2.2 Induction of mitochondrial oxidative stress and lysosomal blockade

Preliminary dose-response experiments were performed to establish optimal conditions for *in vitro* oxidative stress induction using carbonyl cyanide m-chlorophenyl hydrazone (CCCP, Sigma-Aldrich, Cat. #C2759) for all the cell lines. These experiments determined the appropriate sublethal CCCP concentration and exposure duration required to induce mitochondrial oxidative stress while maintaining cell viability >75%. 4T1 cells were treated with mitochondrial impairment agents Valinomycin (50 μM) and CCCP (10 nM) at time intervals of 0, 15, 30, 60 min, 4 h and overnight. Following treatment, cells were lysed using RIPA lysis buffer (Millipore Sigma, Cat. #20188) and lysates were collected. For EV isolation experiments, 4T1 and IOMM-Lee cells were treated with 10 nM CCCP overnight to induce mitochondrial stress. To inhibit lysosome-mitophagosome fusion, cells were simultaneously treated with 50 nM Bafilomycin A1(Baf) (Sigma-Aldrich, Cat. #196000). After 5 days of culture, supernatants were collected and stored for subsequent EV isolation and analysis.

### 2.3 Cell viability, apoptosis, mitochondrial toxicity and reactive oxidative species assays

#### 2.3.1 Cell viability assay

Briefly, 4T1 and IOMM-Lee cells were seeded into 96-well opaque walled plates (Thermo Scientific™ Cat. #265302) at a density of 1,000 cells/well. Cells were treated 24 h later with the vehicle control DMSO (Sigma Aldrich Cat. # 276855), dosages of CCCP (5, 10, 15, 20, 25 nM), constant Baf (50 nM) and their respective combinations overnight at 37°C. The following day, the plates were equilibrated at room temperature for 30 min. Cell viability was then measured by adding CellTiter-Glo^®^ Reagent (Promega, Cat. #G7570) in an amount equal to the volume of the cell culture medium in each well. The contents were mixed on an orbital shaker for 2 min to induce cell lysis, followed by a 10-minute incubation at room temperature to stabilize the luminescent signal. Control wells containing medium alone were set up to measure background luminescence. Luminescence was recorded from each well using a BioTek Cytation 5 Cell Imaging Multimode Reader. Data were normalized to the cell viability of the untreated control cells, which were set as 100%.

#### 2.3.2 Caspase-3/7 apoptosis assay

To assess whether cell death occurred following CCCP and Baf treatment, Caspase-3,7 activity was measured in the 4T1 and IOMM-Lee cells using the Caspase-Glo^®^ 3/7 Assay (Promega Cat. #G8091). 1,000 cells/well were seeded in 96-well opaque walled plates (Thermo Scientific™ Cat. #265302) and treated with the same CCCP and Baf concentrations as described in the *Cell Viability Assay,* above, for 12 h at 37°C. After treatment, the plates were removed from the incubator, and an equal volume (100 μL) of Caspase-Glo^®^ 3/7 reagent was added to each well. The plates were shaken at 300–500 rpm for 30 s and then incubated at room temperature for 2–3 h. Luminescence from each well was then measured, and the extent of apoptosis was determined by comparing the luminescence readings of treated cells to those of untreated cells. Data were normalized to the luminescence of untreated cells.

#### 2.3.3 Mitochondrial toxicity assay

4T1 and IOMM-Lee cells were seeded into opaque-walled 96-well plates, at a density of 1,000 cells/well as in the other assays, and incubated overnight. The following day, the cells were treated with CCCP and Baf as described in the *Cell Viability Assay* for 12 h at 37°C. After treatment, the Mitochondrial ToxGlo™ assay (Promega, Cat. #G8000) was performed according to the manufacturer’s instructions. Approximately 20 µL of assay reagent was added to each well. The plates were gently mixed on an orbital shaker for 2 min to ensure uniform distribution, followed by a 30-minute incubation at room temperature. Fluorescence intensity was then measured at 485 nm Excitation and 520–530 nm Emission using the BioTek Cytation 5 Cell Imaging Multimode Reader to assess the reduction in mitochondrial membrane potential. Following fluorescence measurement, the plates were equilibrated to room temperature for 5–10 min and 100 µL of ATP Detection Reagent was added to each well. The plates were mixed on an orbital shaker at 500–700 rpm for 1–5 min and then incubated for 10 min at room temperature to stabilize the luminescent signal. Final luminescence readings, indicative of overall ATP activity, were recorded. Mitochondrial cytotoxicity and ATP activity were evaluated by comparing the fluorescence and luminescence signals from treated wells to those of control wells, respectively. Data were normalized to the fluorescence and luminescence intensities of untreated cells.

#### 2.3.4 Reactive oxidative species (ROS) assay

Intracellular ROS levels were analyzed using the Cellular ROS Detection Assay Kit (Abcam, Cat. #ab139473) according to the manufacturer’s protocol. For the fluorescence microplate assay, cells were seeded at a density of 5,000 cells/mL in a 96-well plate and incubated overnight under standard culture conditions. The following day, cells were treated with the ROS detection mix in a sufficient volume to cover the cell monolayer and incubated under normal cell culture conditions for 2 h. After incubation, the detection mix was carefully removed, and the cells were washed with 1X wash buffer provided by the kit. Cells were then treated with CCCP (10 nM), Baf (50 nM), and combination of both (CCCP 10nM; Baf 50 nM) for three different durations: 30 min, 60 min, and overnight. Positive control wells were treated with Pyocyanin (350 µM), while negative control wells were treated with N-acetyl-L-cysteine (5 mM) for 30 min to achieve the desired ROS levels, as per the manufacturer’s instructions. After treatment, cells were washed twice with 1X wash buffer, and fluorescence intensity was measured using the BioTek Cytation 5 Cell Imaging Multimode Reader at 485 nm Excitation and 525 nm Emission wavelengths. Measured raw fluorescence intensity were used to analyze the three different treatment groups at each timepoint.

### 2.4 EV isolation by differential ultracentrifugation and nanoparticle tracking analysis

Both treated and untreated 4T1 and IOMM-Lee cell cultures were grown for 5 days at 37°C in a 5% CO₂ atmosphere. The culture supernatants were collected and initially centrifuged at 500 × g for 10 min to remove cell debris. The resulting supernatant was then centrifuged first at 2,000 × g for 45 min at 4°C and the collected EV pellet was labeled as “2 K”, and the remaining supernatant labelled as 2 K+ (which include the 10K and 100 K EV populations) and stored at 4°C for subsequent downstream assays. Nanoparticle tracking analysis (NTA) of isolated EVs was performed to determine the concentrations of EVs using the ZetaView^®^ Twin (Particle Metrix) and the corresponding software ZetaView version 8.06.01 (Théry et al., 2018).

### 2.5 Western blot analysis

Whole-cell extracts (10 μg) and concentrated differential ultracentrifugation EV samples (10 μL) were resuspended in 10 μL of Laemmli buffer, heated at 95°C for 3 min, and loaded onto a 4%–20% Tris-Glycine SDS gel (Invitrogen). The gels were run at 150 V, then transferred to polyvinylidene difluoride (PVDF) membranes (Millipore Sigma) via wet transfer at 25 V for 2 h at room temperature. Membranes were blocked in 5% milk in PBS-1X with 0.1% Tween 20 (PBST) for 30 min at 4°C. The membranes were incubated overnight at 4°C with the appropriate primary antibody, diluted in PBST according to the manufacturer’s recommendations. Antibodies used included anti-CD81 (Cell Signaling, Cat. # ab79559), anti-β-Actin (Cell Signaling, Cat. # 4970 S), anti-PINK1 (Cell Signaling, Cat. # 6946 S), and anti-calnexin (Cell Signaling, Cat. # 2679 T). After incubation, membranes were washed three times with PBST for 5 min each and then incubated with the appropriate horseradish peroxidase (HRP)-conjugated secondary antibodies (Jackson ImmunoResearch, AffiniPure™, Cat. # AB_2313567) for an hour at room temperature. Detection was performed using SuperSignal™ West Dura Extended Duration Substrate (Thermo Scientific™, Cat. # 34075), and luminescence was visualized using the Azure Biosystems 400. Western blot band pixel intensity was quantified using ImageJ software, with measurements taken in triplicate.

### 2.6 Statistical analysis

Statistical Analyses were performed using GraphPad Prism 9 Software using default settings unless otherwise noted. Appropriate analyses are described within Figure legends.

## 3 Results

### 3.1 Secretory mitophagy promotes cell survival under oxidative stress

**FIGURE 1:**
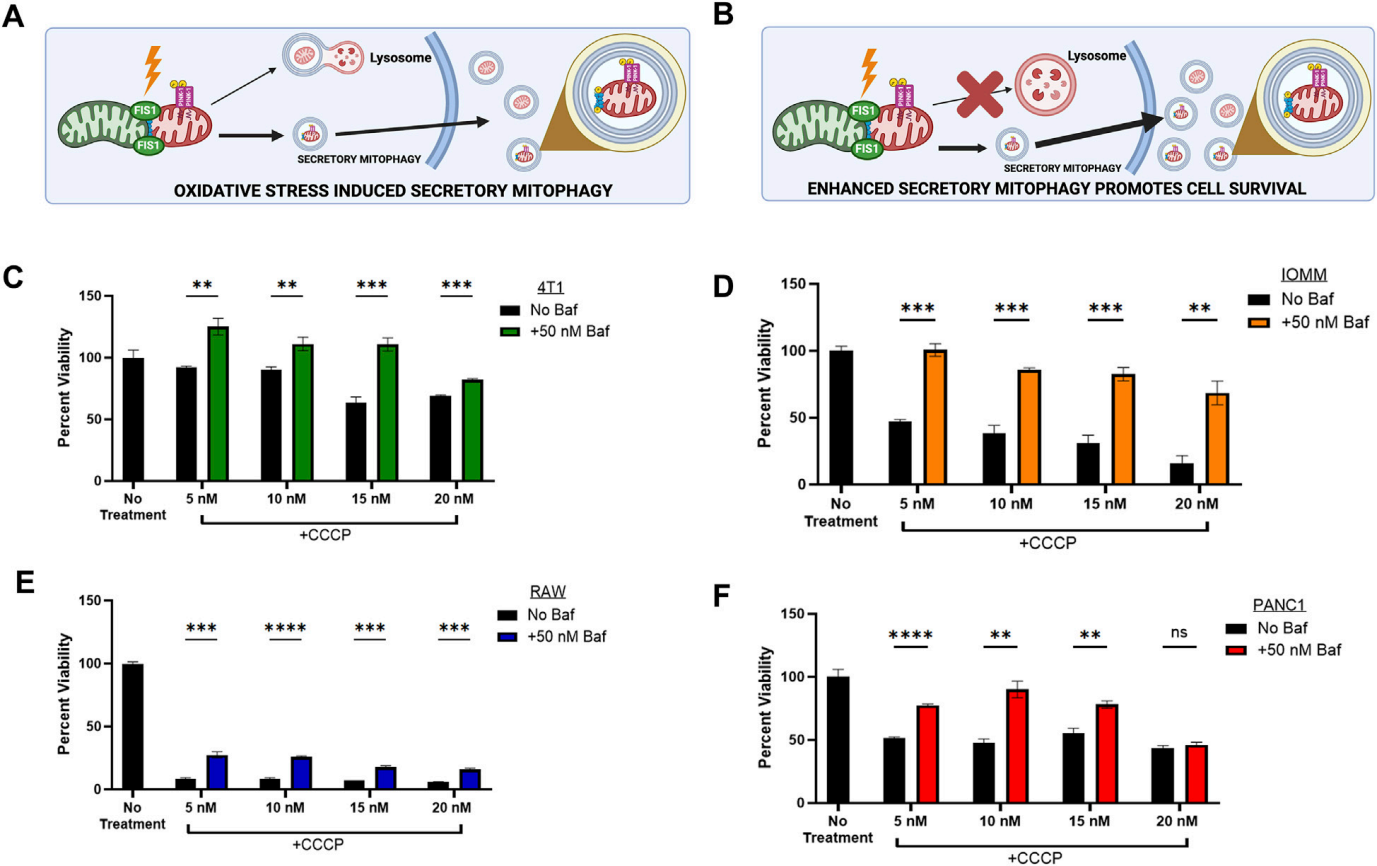
| Secretory Mitophagy promotes cell survival. **(A)** Oxidative stress induces export of EVs derived from cellular mitophagy. **(B)** The shift to secretory mitophagy can be enhanced via lysosomal blockade. Mitophagy induction via CCCP reduces cell viability. Cellular viability is rescued upon combination treatment of lysosome-autophagosome fusion inhibitor Baf (50 nM) and CCCP within **(C)** Murine triple-negative breast cancer 4T1 cells, **(D)** Meningioma IOMM-Lee cells, **(E)** Murine undifferentiated macrophage RAW 264.7 cells, and **(F)** Pancreatic epithelial carcinoma PANC1 cells. Statistics: Error bars represent the standard deviation. p-values calculated via unpaired *t*-test with correction for multiple comparison using the Holm-Sidak method. ns, not significant (p > 0.05); *p ≤ 0.05; **p ≤ 0.01; ***p ≤ 0.001; ****p ≤ 0.0001.

Cancer progression is a complex, multi-step process in which cancer cells face both high energy demands and elevated oxidative stress simultaneously. Maintaining a balance between mitochondrial biogenesis and the removal of damaged mitochondria is essential (Arfin et al., 2021). Mitophagy, the selective degradation of dysfunctional mitochondria, helps maintain mitochondrial health, but is relatively slow, taking 8–18 h (Kubli and Gustafsson, 2012). Under oxidative stress, mitophagy may become overwhelmed, resulting in an accumulation of autophagosomes that cannot be cleared through lysosomal degradation alone (Figure 1A). Secretory mitophagy likely offers a faster alternative by exporting damaged mitochondria to support cell survival (Figure 1B). However, the mechanisms that trigger the switch from lysosomal degradation to secretory mitophagy remain unclear. The presence of PINK1 + EVs serves as a marker of secretory mitophagy activation (Howard et al., 2022). PINK1-mediated mitophagy can be induced by CCCP (carbonyl cyanide m-chlorophenyl hydrazone) and valinomycin (Jin and Youle, 2012). CCCP, a mitochondrial uncoupler, disrupts the proton gradient by shuttling protons across the inner mitochondrial membrane, leading to loss of membrane potential (Park et al., 2018). In contrast, valinomycin, is a potassium ionophore that selectively transports K^⁺^ ions across the membrane, collapsing the mitochondrial membrane potential without directly affecting proton transport (Fedorowicz et al., 2014). Following a single treatment of murine triple-negative 4T1 cells with CCCP (10 nM) and valinomycin (50 μM), we observed that CCCP induced a transient, time-dependent mitochondrial depolarization, marked by increased intracellular PINK1 expression peaking at 30–45 min, followed by a decrease after 45 min. In contrast, valinomycin caused sustained mitochondrial depolarization, with persistently high intracellular PINK1 expression, indicating irreversible damage (Supplementary Figure S1). These findings highlight the reversible effects of CCCP compared to the permanent effects of valinomycin, establishing CCCP as a sublethal oxidative stress inducer suitable for our study.

To investigate the mechanisms of secretory mitophagy, mitochondrial depolarization was induced in the following cell lines: murine triple-negative breast cancer (4T1), human meningioma (IOMM-Lee), human epithelial pancreatic carcinoma (PANC1), and murine undifferentiated macrophages (RAW 264.7) using CCCP. This resulted in a dose-dependent decrease in cell viability compared to untreated controls (Figures 1C–F). To force the elimination of damaged mitochondria through secretory mitophagy, we selectively blocked autophagosome-lysosome fusion through treatment with Bafilomycin A1 (Baf). Baf disrupts lysosomal acidification, induces G1 cell cycle arrest (Yan et al., 2016; Shacka et al., 2006), and reduces mitochondrial-lysosome colocalization (Klionsky et al., 2008; Yamamoto et al., 1998; Dröse and Altendorf, 1997; Liang et al., 2023). Combining CCCP with Baf stimulated secretory mitophagy by preventing lysosome-dependent degradation of damaged mitochondria. In our study, Baf significantly rescued cell viability (p < 0.05–0.001) across all cell lines treated with CCCP (Figures 1C–F). The strongest effect was observed within IOMM-Lee cells, where Baf addition reversed the ∼50% reduction in cell viability caused by CCCP alone (Figure 1D). In RAW 264.7 macrophages, the combination treatment significantly improved cell viability compared to CCCP alone (p < 0.005), although overall cell viability remained below 75% of untreated levels (Figure 1E). This observation aligns with evidence that oxidative stress suppresses mitophagy in macrophages to support phagocytosis (Patoli et al., 2020). Overall, these findings indicate that stimulating secretory mitophagy through lysosomal blockade enhances cell viability during oxidative stress, providing a critical survival mechanism for cancer cells under oxidative challenges.

### 3.2 Effect of secretory mitophagy on mitochondrial health, apoptosis, and ROS generation

**FIGURE 2:**
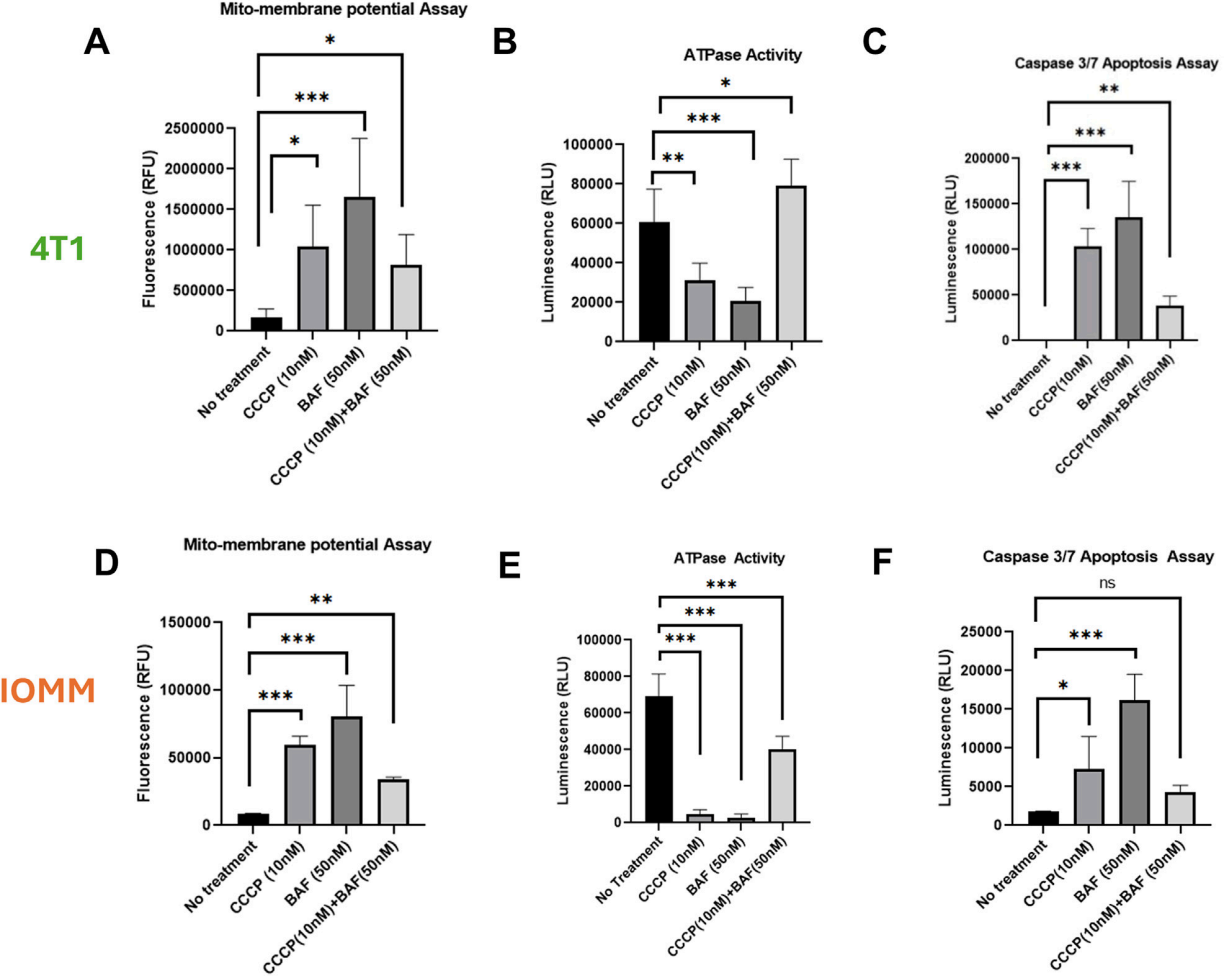
Characterization of 4T1 **(A–C)** and IOMM-Lee **(D–E)** cells lines for mitochondrial health and apoptotic induction. Using sublethal concentrations of CCCP (10 nM) and Baf (50 nM) or their combination, the mitochondrial membrane depolarization **(A, D)** is significantly greater with single treatment, particularly with Baf, but is rescued by the combination of CCCP and Baf. ATPase activity **(B, E)** is increased, and caspase 3/7 activity **(C, F)** is reduced during combination therapy compared to individual treatments. Statistics: Error bars represent the standard deviation. p-values were calculated by ordinary one-way ANOVA for multiple comparisons using the two-stage step-up method of Benjamini, Krieger, and Yekutieli. Ns = not significant (p > 0.05); *p ≤ 0.05; **p ≤ 0.01; ***p ≤ 0.001; ****p ≤ 0.0,001.

Secretory mitophagy protects cells from oxidative stress; however, its functional implications for overall intracellular mitochondria health remain unexplored. To assess intracellular mitochondrial health following the stimulation of secretory mitophagy, we measured mitochondrial toxicity (via mitochondrial membrane potential), mitochondrial ATPase activity, and apoptosis (caspase-3/7 activation) in 4T1 and IOMM-Lee cells treated with CCCP, Baf, or their combination. Both CCCP and Baf monotherapies increased mitochondrial toxicity, as indicated by higher levels of membrane depolarization (Figures 2A, D). In contrast, combination treatment significantly reduced mitochondrial toxicity compared to either treatment alone (p < 0.05–0.01). ATPase activity showed an inverse trend, with combination therapy rescuing ATP production (Figures 2B, E). Specifically, in 4T1 cells, combination treatment increased ATPase activity beyond the untreated controls (p < 0.05). In IOMM-Lee cells, combination treatment restored ATP production to approximately half of untreated control levels, mitigating the impairment caused by monotherapies (p < 0.001). Apoptosis, measured by caspase-3/7 activation, was highest with Baf monotherapy application (p < 0.05–0.001), whereas combination treatment reduced apoptosis close to untreated control levels (p ≤ ns-0.01) (Figures 2C, F). Another key indicator of intracellular toxicity is ROS generation, which is often associated with high energetic stress. To assess this, we measured ROS levels in 4T1 cells treated with CCCP, Baf and their combination at various time points (30 min, 60 min, and overnight). Across all time points, ROS levels did not differ significantly compared to untreated controls (Supplementary Figure S2). Minimal ROS generation was observed, consistent with previous reports indicating that higher doses of CCCP (e.g., 10 µM) are required to induce significant ROS production (Xiao et al., 2017). Our findings suggest that the intracellular mitochondrial health is not adversely affected by secretory mitophagy. Collectively, these results demonstrate that secretory mitophagy, induced by CCCP and Baf co-treatment, improves intracellular mitochondrial function and reduces apoptosis, highlighting its potential role in cellular survival under oxidative stress.

### 3.3 PINK1+ EV content is enhanced by secretory mitophagy

**FIGURE 3:**
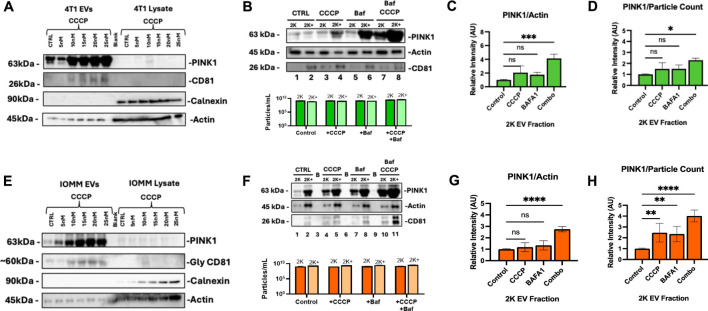
PINK1 EV export correlates with Secretory Mitophagy induction under oxidative stress. **(A)** CCCP treatment in 4T1 cells increases PINK1+/CD81 + EV secretion while maintaining low cytoplasmic PINK1 levels. Calnexin is detected only in cell lysates and not in EVs. **(B)** *Upper panel:* Western blot demonstrates elevated PINK1 levels in 4T1 EVs with combination treatment. *Lower panel:* Total EV particle counts (2 and 2 K+) remain unaffected regardless of treatment. **(C, D)** Western blot quantification and Zeta-View nanoparticle tracking analysis of 2 K EVs reveal a significant increase in PINK1/Actin and PINK1/particle ratios for combination therapy compared to monotherapy, respectively. **(E)** In IOMM-Lee cells, CCCP induces PINK1+/CD81 + EV secretion while cytoplasmic PINK1 levels remain consistently low. **(F)** *Upper panel*: Western blot reveals elevated PINK1 in IOMM-Lee EVs with combination treatment. *Lower panel:* Total EV counts (2 and 2 K+) remain unaffected by mitophagy stimulation and CCCP-Baf co-treatment. **(G, H)** Combination treatment increased PINK1/Actin intensity and PINK1/particle ratios within IOMM-Lee 2 K EVs. Statistics: Quantifications were based on two biological replicates for 4T1 cells and two for IOMM-Lee cells. Error bars represent standard deviation. p-values were calculated using two-way ANOVA with Dunnett’s multiple comparisons test. ns, not significant (p > 0.05); *p ≤ 0.05; **p ≤ 0.01; ***p ≤ 0.001; ****p ≤ 0.0001.

Tumor cells export PINK1-containing EVs under oxidative stress, signaling the activation of secretory mitophagy. We hypothesize that inducing secretory mitophagy increases the proportion of PINK1+ EVs within the total EV population. Sublethal CCCP treatment (5–20 nM) induces oxidative stress, leading to an increase in PINK1+ EVs, while cytosolic PINK1 levels remained low (Figures 3A, E). This observation aligns with PINK1’s role in localizing to damaged mitochondria, to initiate mitophagy. On the other hand, in undamaged mitochondria, PINK1 is continuously degraded via mitochondrial inner membrane enzymes (Lazarou et al., 2015; Deas et al., 2011; Pollock et al., 2021; Gonçalves and Morais, 2021; Becker et al., 2012). Under oxidative stress, we observed an increased export of PINK+/CD81 + EVs, indicating the utilization of secretory mitophagy to clear damaged mitochondria. Calnexin, an endoplasmic reticulum marker and negative control for EV contamination (Samaeekia et al., 2018), was detected only in cell lysates, confirming the purity of EV isolation. The isolated EV populations were categorized as “2 K”—EVs collected from the culture supernatant after centrifugation at 2,000 × g for 45 minutes—and “2 K+”, representing the remaining supernatant containing other EV populations. We focused on 2 K EVs due to their origin and secretion pathways. The 2 K fraction likely to consists of multi-vesicular bodies (MVBs), which play a critical role in trafficking cellular debris; these MVBs can either fuse with the plasma membrane to release exosomes or fuse with the lysosome for degradation (Veerabhadraswamy et al., 2024). Our analysis revealed that total EV particle counts (2 and 2 K+) were unaffected by the treatment with CCCP, Baf, or their combination (Figures 3B, F, lower panels). Both 4T1 and IOMM-Lee cells demonstrate increased PINK1 expression when under combination treatment (Figures 3B, F, upper panels). The combination treatment consequently increased the extracellular PINK1 levels independent of total protein (Actin) and particle count (Figures 3C, D, G, H). In particular, 4T1 cells had a significant increase in PINK1 content (PINK1/Actin, P < 0.001; PINK1/particle count, P < 0.05). These findings suggest that PINK1 + EVs serve as highly sensitive readouts of ongoing intracellular mitophagy.

## 4 Discussion

Mitochondrial-associated vesicle shedding, a primordial process predating lysosomal and peroxisomal systems, is essential for maintaining mitochondrial quality by removing damaged mitochondria (Gould et al., 2016; McBride, 2018). Under oxidative stress, cells export damaged mitochondria through extracellular vesicles, a process further enhanced when lysosomal degradation is inhibited. Our previous work identified PINK1 + EVs as a key marker of secretory mitophagy. PINK1 orchestrates this process by marking depolarized mitochondria for removal. In its phosphorylated form, PINK1—along with the translocase of the outer mitochondrial membrane 20 (TOM20) and other mitophagy-specific proteins resided in the proteomic repertoire within EVs (Howard et al., 2022). Our current findings reinforce this model, demonstrating a positive correlation between oxidative stress levels, oxidative phosphorylation demands, and PINK1+ EV export. Blocking lysosomal function with Baf further amplified the release of PINK1 + EVs, suggesting that secretory mitophagy acts as an alternative pathway when canonical mitophagy becomes overwhelmed.

Tumor cells utilizing this pathway exhibit a marked survival advantage under oxidative stress. Our findings reveal that increased PINK1 + EV secretion correlates with improved cell viability, enhanced ATP production, and reduced intracellular mitochondrial toxicity. This finding was generalized across murine and human cancer cell lines. In some cases, the viability was rescued by greater than 75% of the cells subjected to oxidative stress (Figure 1). These results suggest that secretory mitophagy may play a critical role in cancer progression, particularly under oxidative, metabolic, therapeutic, or hypoxic stress.

**FIGURE 4:**
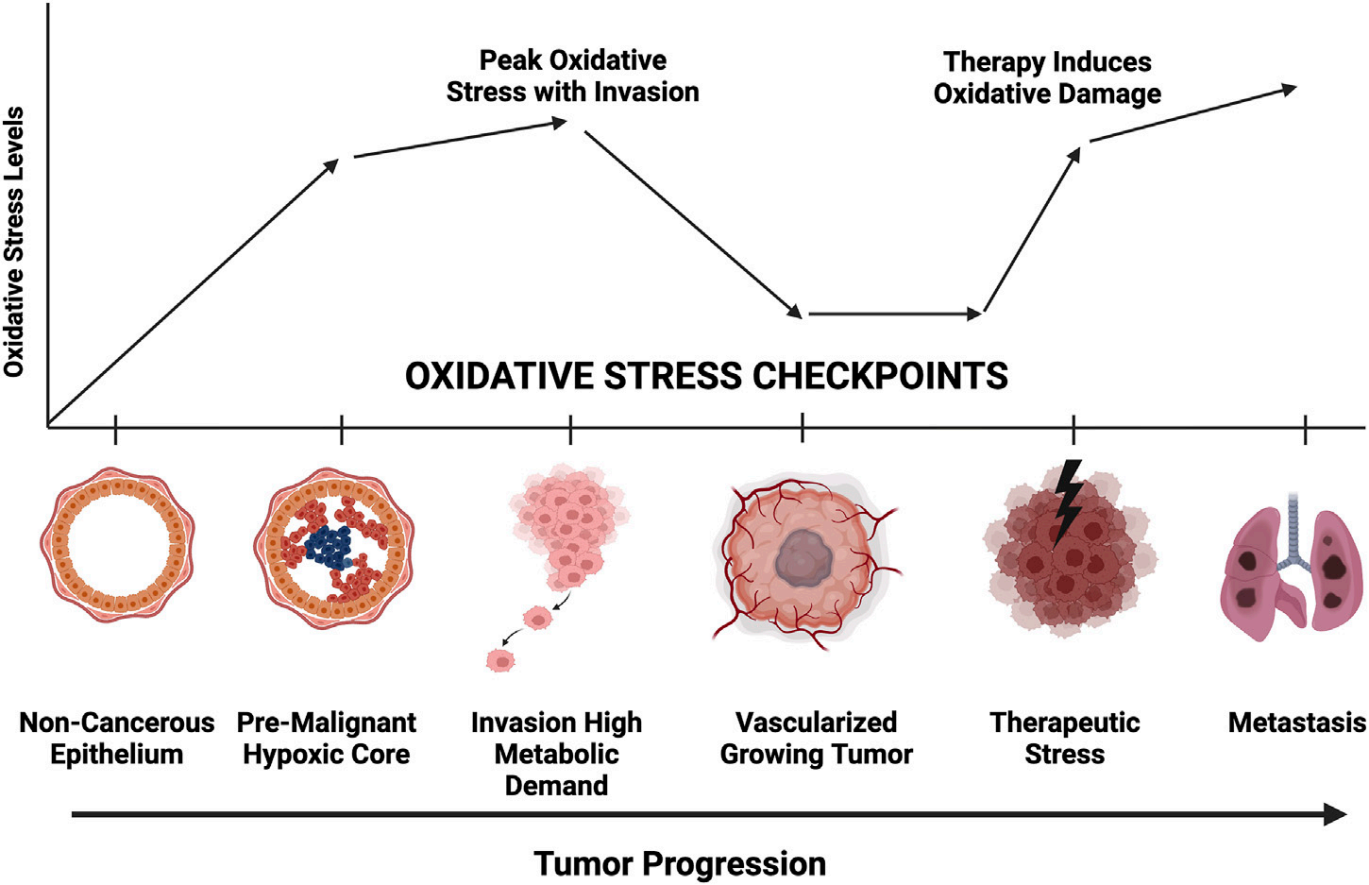
Representative diagram of oxidative stress requirements at each stage of tumor progression. Greater mitochondrial fitness is necessary to withstand hypoxic, nutrient deprivation, invasion, chemotherapeutic challenge, and metastatic colonization. Secretory mitophagy likely facilitate greater tumor cell survival at each checkpoint.

Cancer cells adapt to external challenges by altering metabolic pathways such as glycolysis and oxidative phosphorylation (Schiliro and Firestein, 2021). However, the mechanisms that enable cancer cell survival under oxidative stress, especially through mitophagy and secretory mitophagy, remain underexplored as potential therapeutic targets. Our findings emphasize the role of secretory mitophagy in helping cancer cells manage oxidative stress during various stages of progression (Deepak et al., 2024; Figure 4). Oxidative stress presents challenges at multiple checkpoints in cancer progression, including hypoxia, nutrient deprivation, genotoxic stress in premalignant lesions, heightened metabolic demands during invasion, and damage from therapies such as chemotherapy or radiotherapy. Exporting damaged mitochondria via EVs may be critical for cellular adaptation during these phases. Targeting secretory mitophagy could disrupt this adaptive response, hindering cancer cell survival and progression. A promising therapeutic strategy may involve preventing the transition from canonical mitophagy to secretory mitophagy. Specifically, inhibiting the EV-mediated export of damaged mitochondria offers a novel avenue to interfere with the cancer cell mechanisms that support survival under oxidative stress. These insights lay the groundwork for developing interventions aimed at improving cancer treatment outcomes by mitigating cellular adaptations that promote tumor progression.

## Data Availability

The raw data supporting the conclusions of this article will be made available by the authors, without undue reservation.

## Supplementary material

SUPPLEMENTARY FIGURE S1 Time-dependent Mitochondrial Membrane Depolarization by CCCP (10 nM) and Valinomycin (50 μM), determined by PINK1 Expression. CCCP exhibits a time-dependent mitochondrial depolarization effect, with gradual depolarization observed between 30-45 min, followed by a reversal of the effect after 45 min. In contrast, Valinomycin induces permanent mitochondrial damage, resulting in sustained depolarization regardless of time. PINK1 expression highlights these distinct mechanisms, emphasizing the transient depolarization by CCCP compared to the irreversible impact of Valinomycin.

SUPPLEMENTARY FIGURE S2 4T1 cells treated with CCCP (10 nM), Baf (50 nM), and their combination (CCCP, 10 nM ; Baf 50 nM) at different time points (30 min, 60 min, and overnight) exhibit minimal ROS generation showing a nonsignificant elevation at CCCP low dose (10 nM) confirming the sublethal effects of CCCP. ROS production is higher with monotherapy but is reduced with the combination treatment across all time points. This consistent trend indicates that the treatments do not induce a significant ROS stress response instead stimulating the mitochondrial depolarization and secretory mitophagy subsequently.
