# Supplementary figures and images for: Secretory mitophagy: an extracellular vesicle-mediated adaptive mechanism for cancer cell survival under oxidative stress
Source: Front Cell Dev Biol. 2025 Jan 30;12:1490902. doi: 10.3389/fcell.2024.1490902 (PMC11821619; doi:10.3389/fcell.2024.1490902)

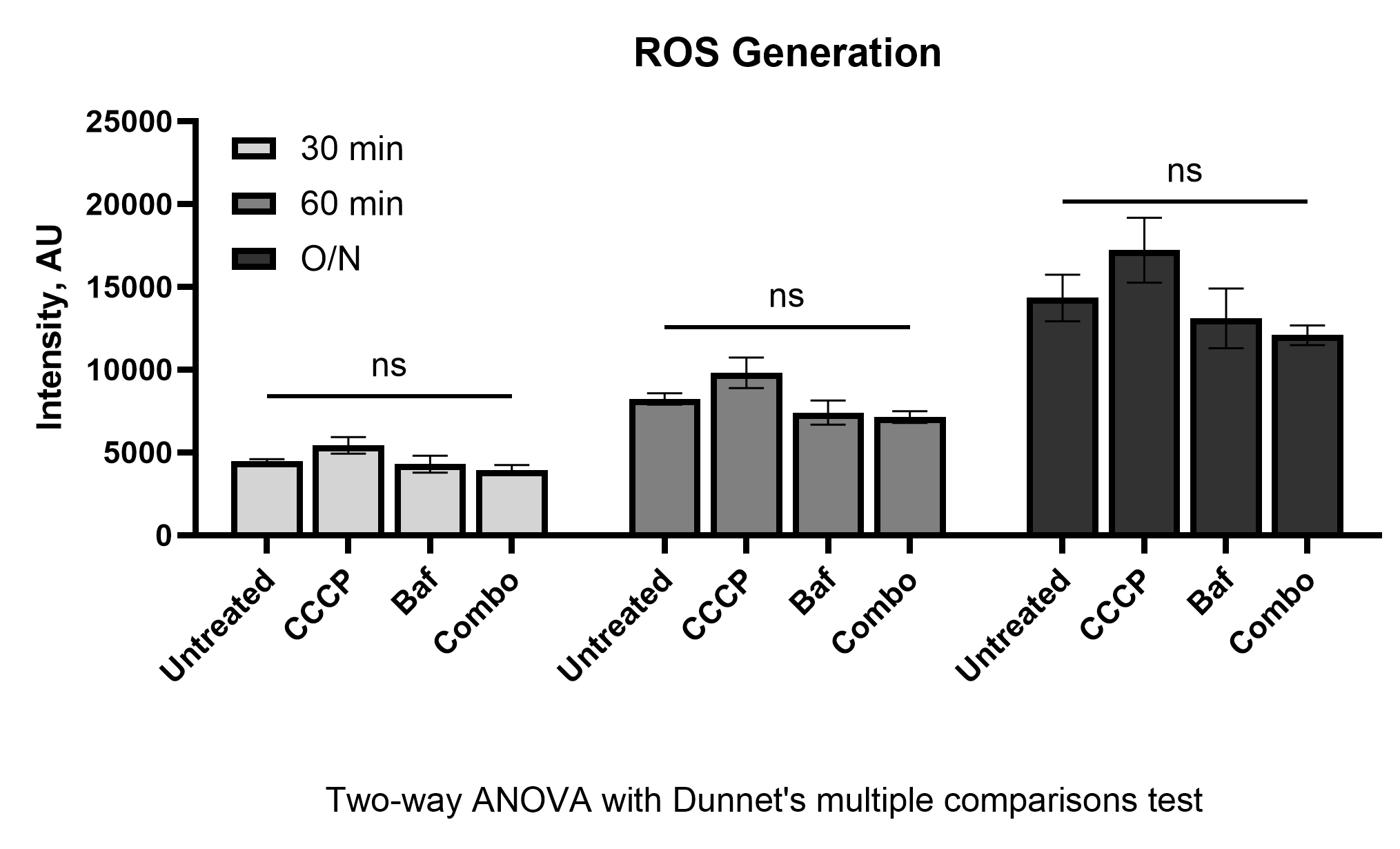

Supplement: Supplementary file 1 [file Image2.tif]

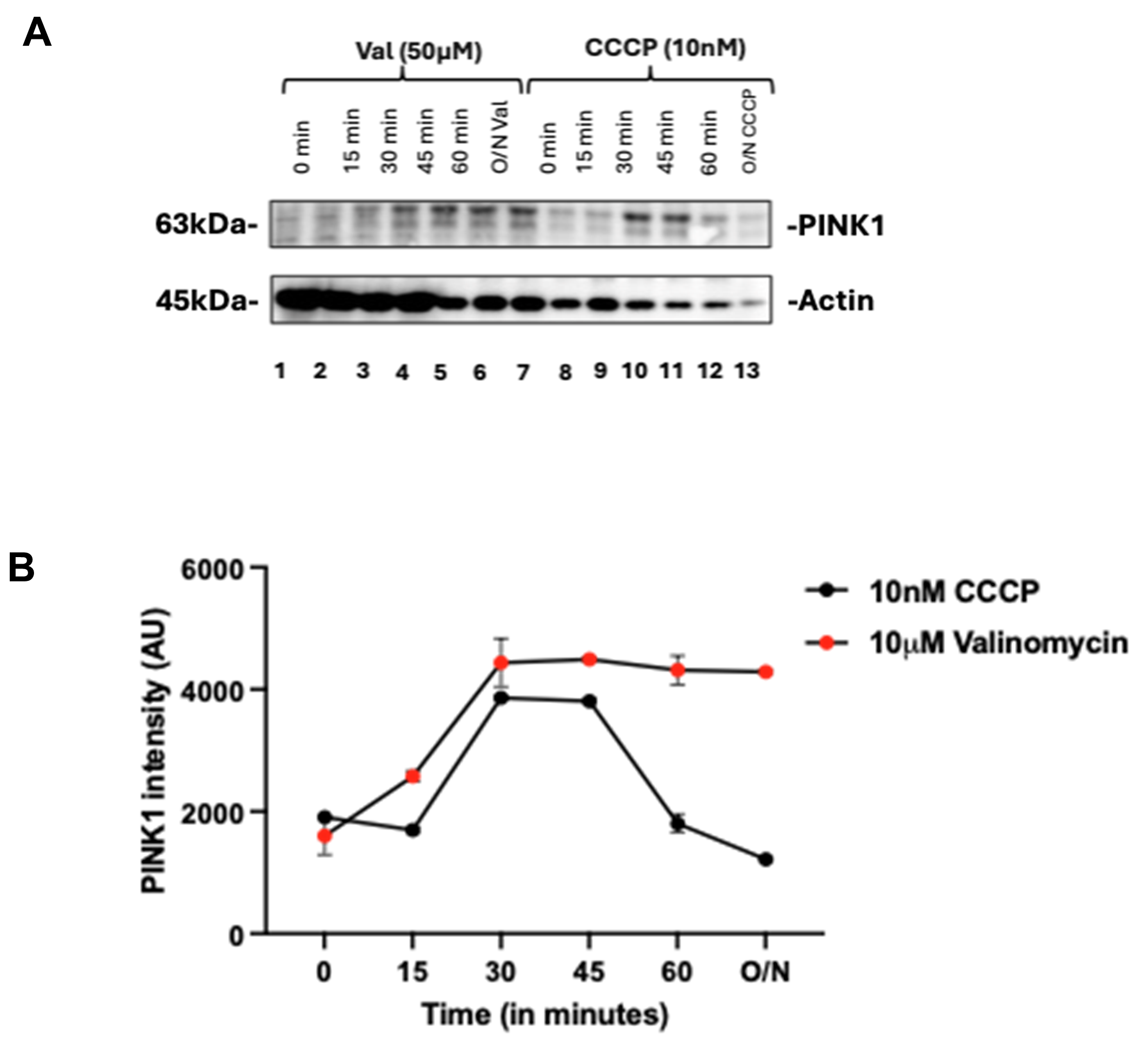

Supplement: Supplementary file 2 [file Image1.tif]
