# Supplementary material for: Secretory mitophagy: an extracellular vesicle-mediated adaptive mechanism for cancer cell survival under oxidative stress
Source: Front Cell Dev Biol. 2025 Jan 30;12:1490902. doi: 10.3389/fcell.2024.1490902 (PMC11821619; doi:10.3389/fcell.2024.1490902)

4T1 dose response -PINK1

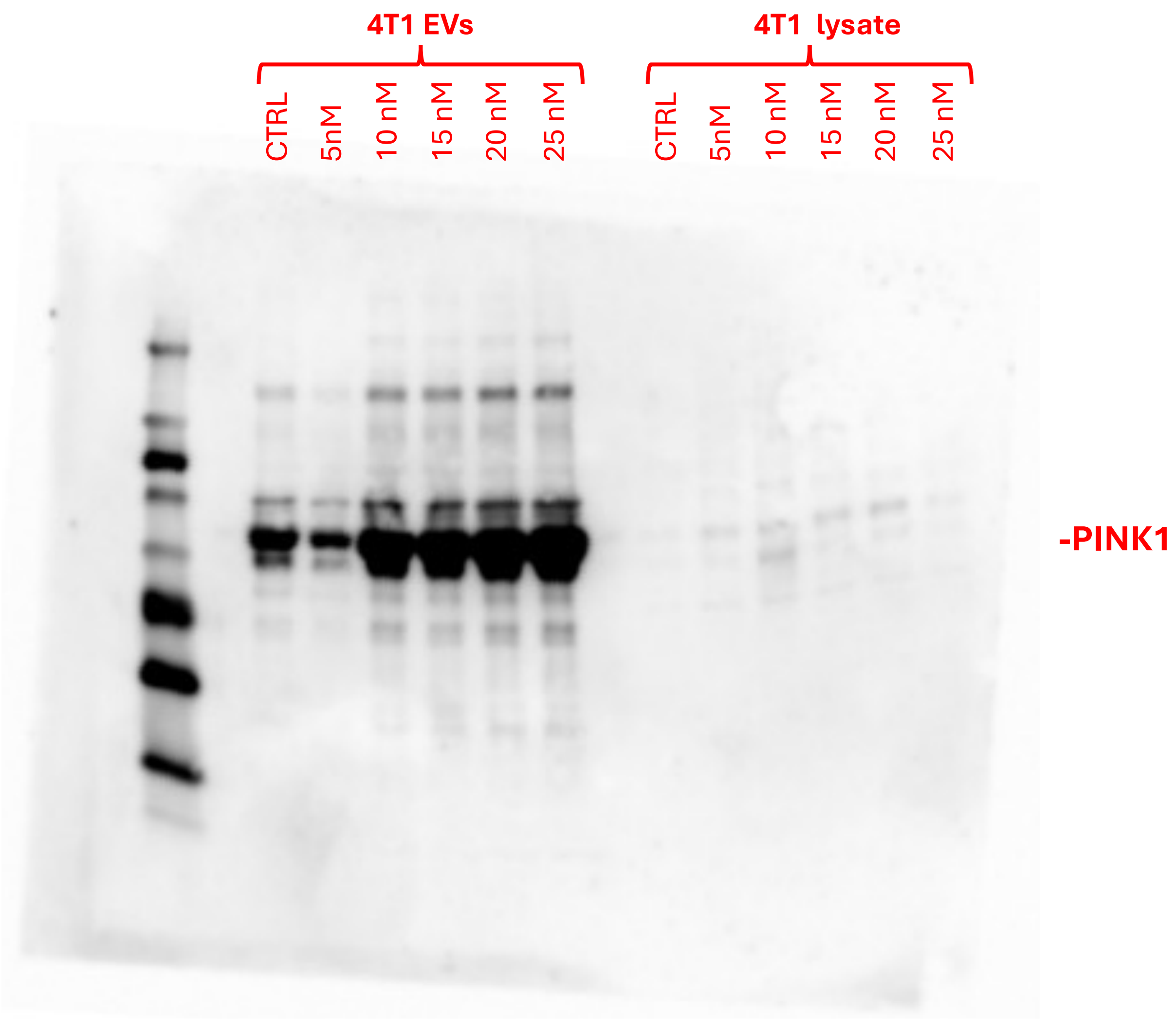

Full western blot image presented in Figure 3A

4T1 dose response- CD81

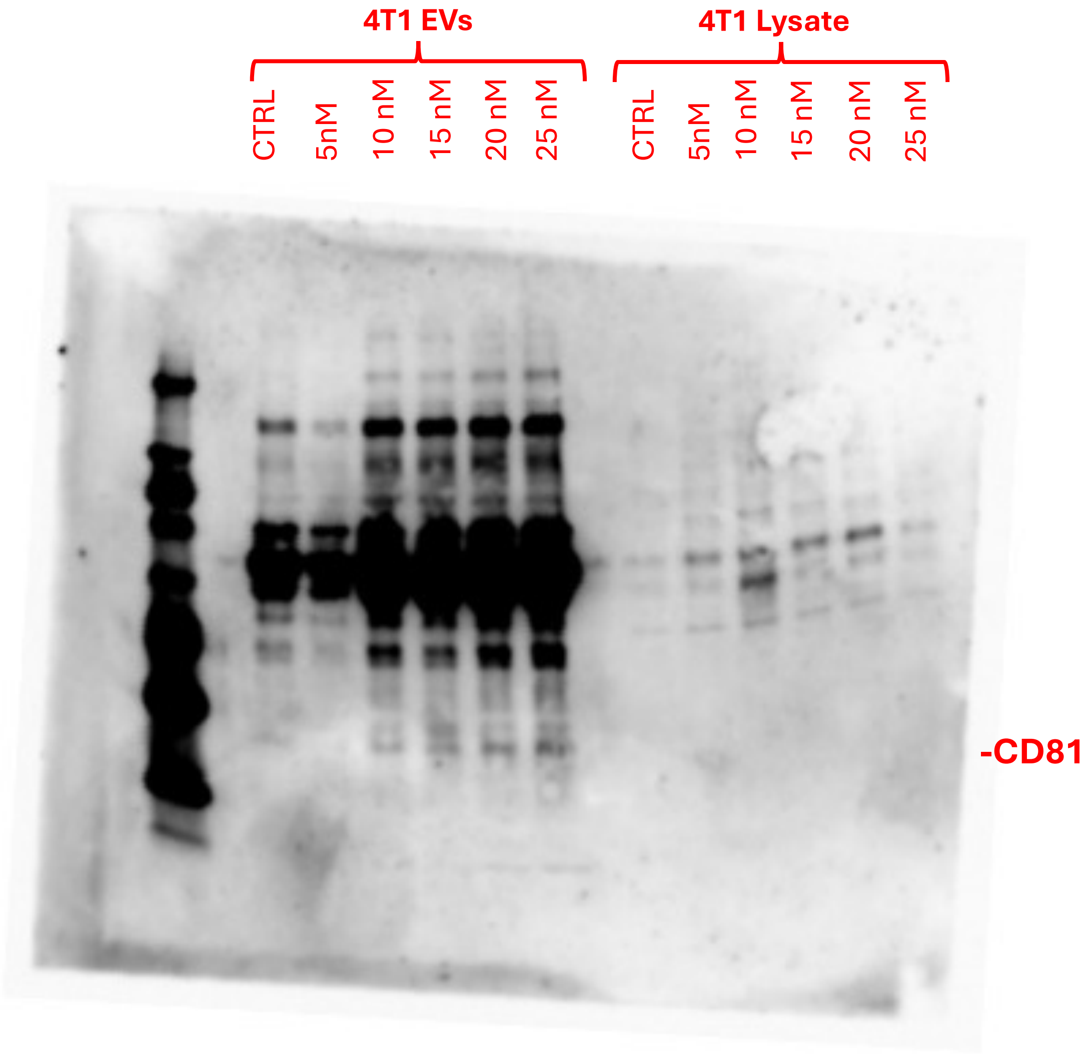

Full western blot image presented in Figure 3A

4T1 dose response-Calnexin

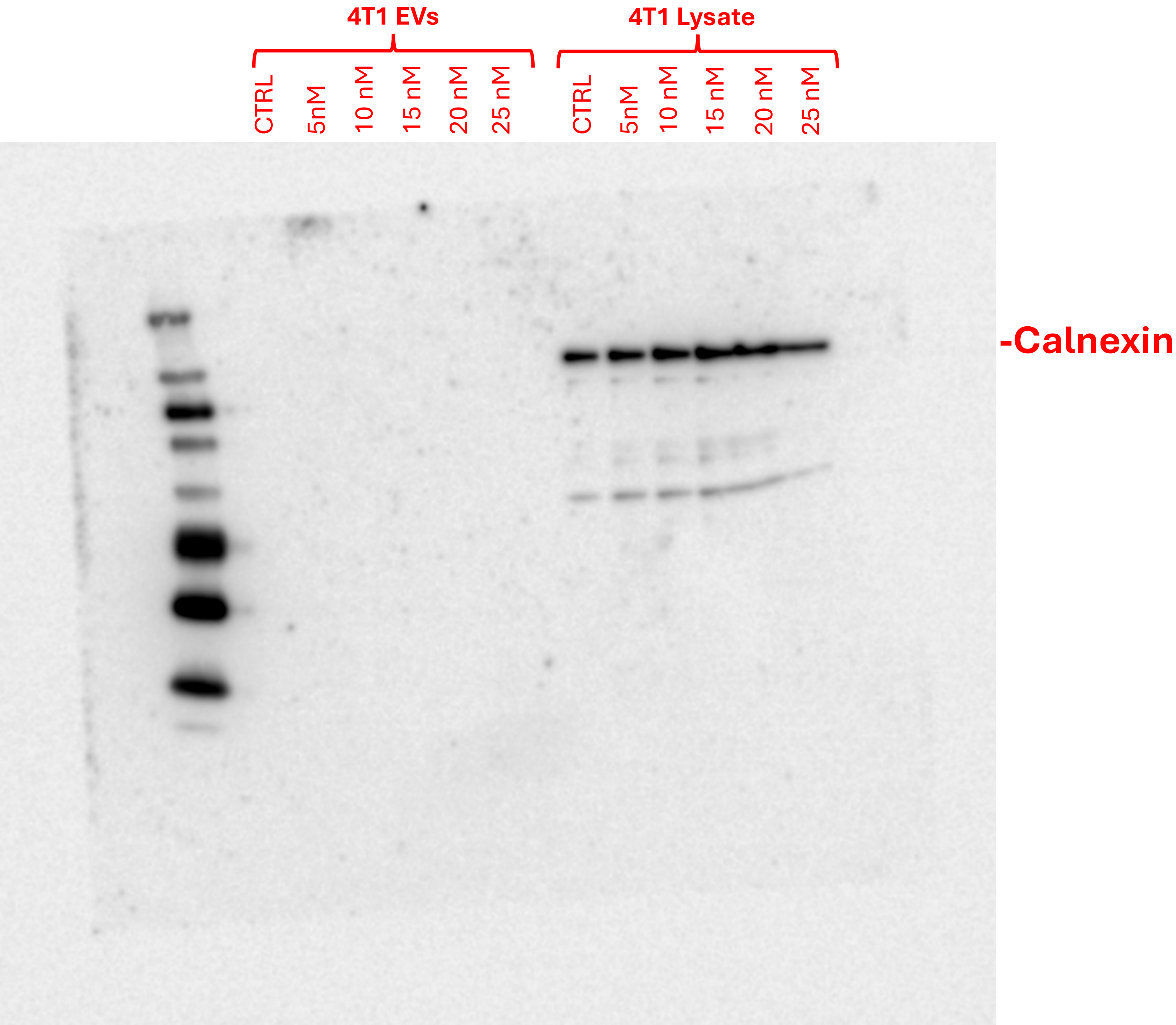

Full western blot image presented in Figure 3A

4T1 dose response-Actin

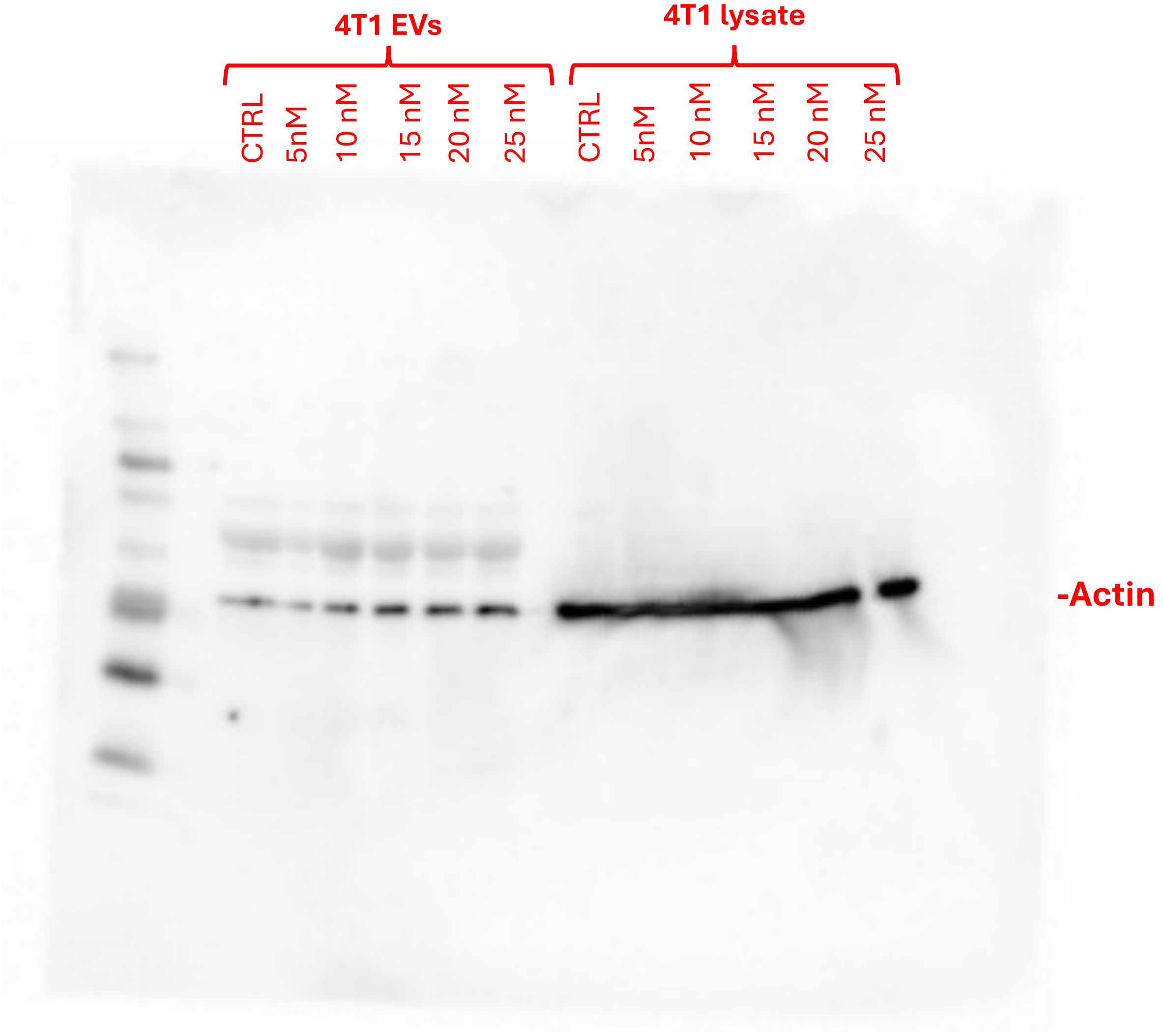

Full western blot image presented in Figure 3A

4T1 treatment PINK1

(Trial 1)

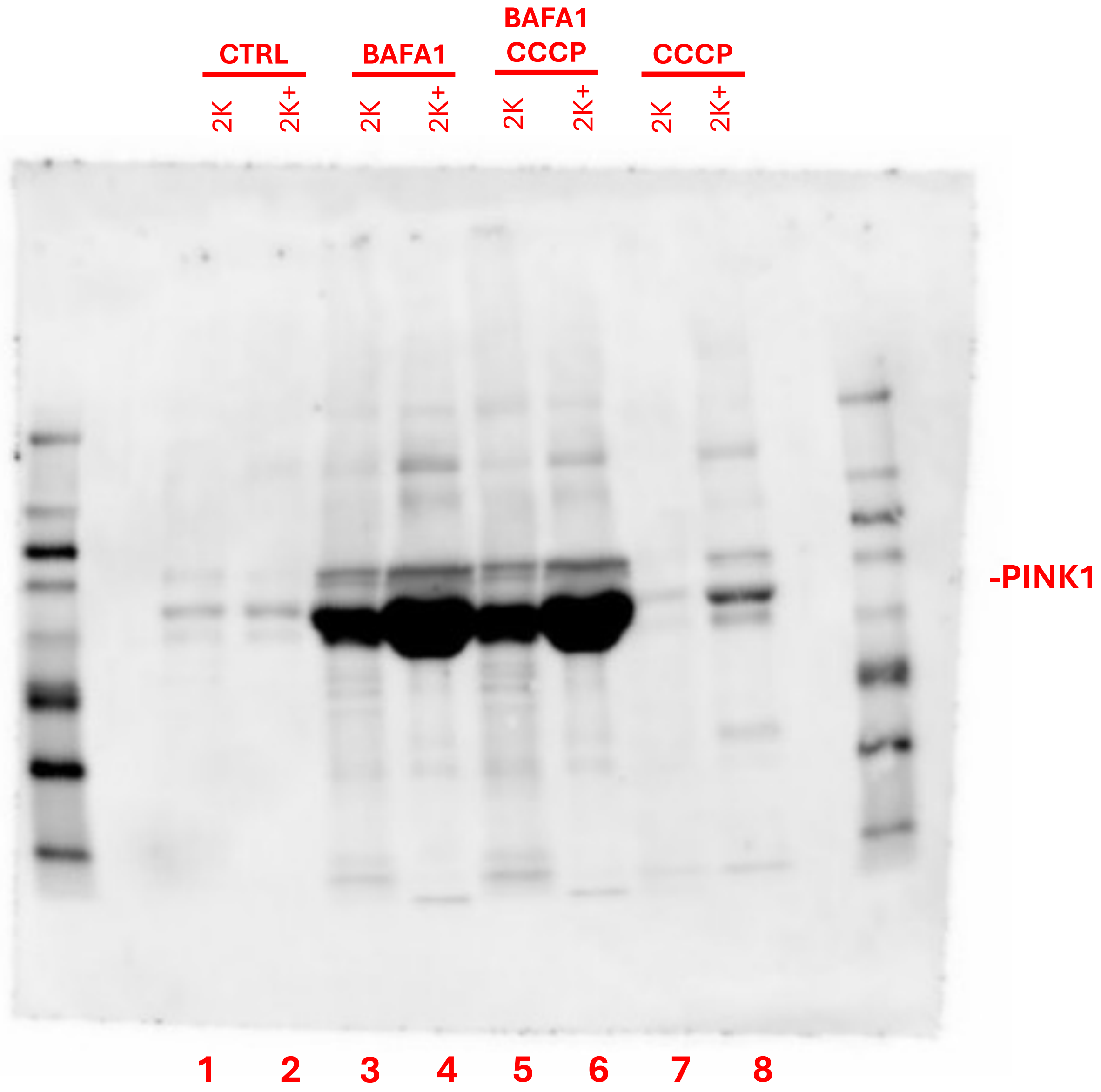

Biological replicate 1 for western blot (PINK1) presented in Figure 3B

4T1 treatment PINK1

(Trial 2)

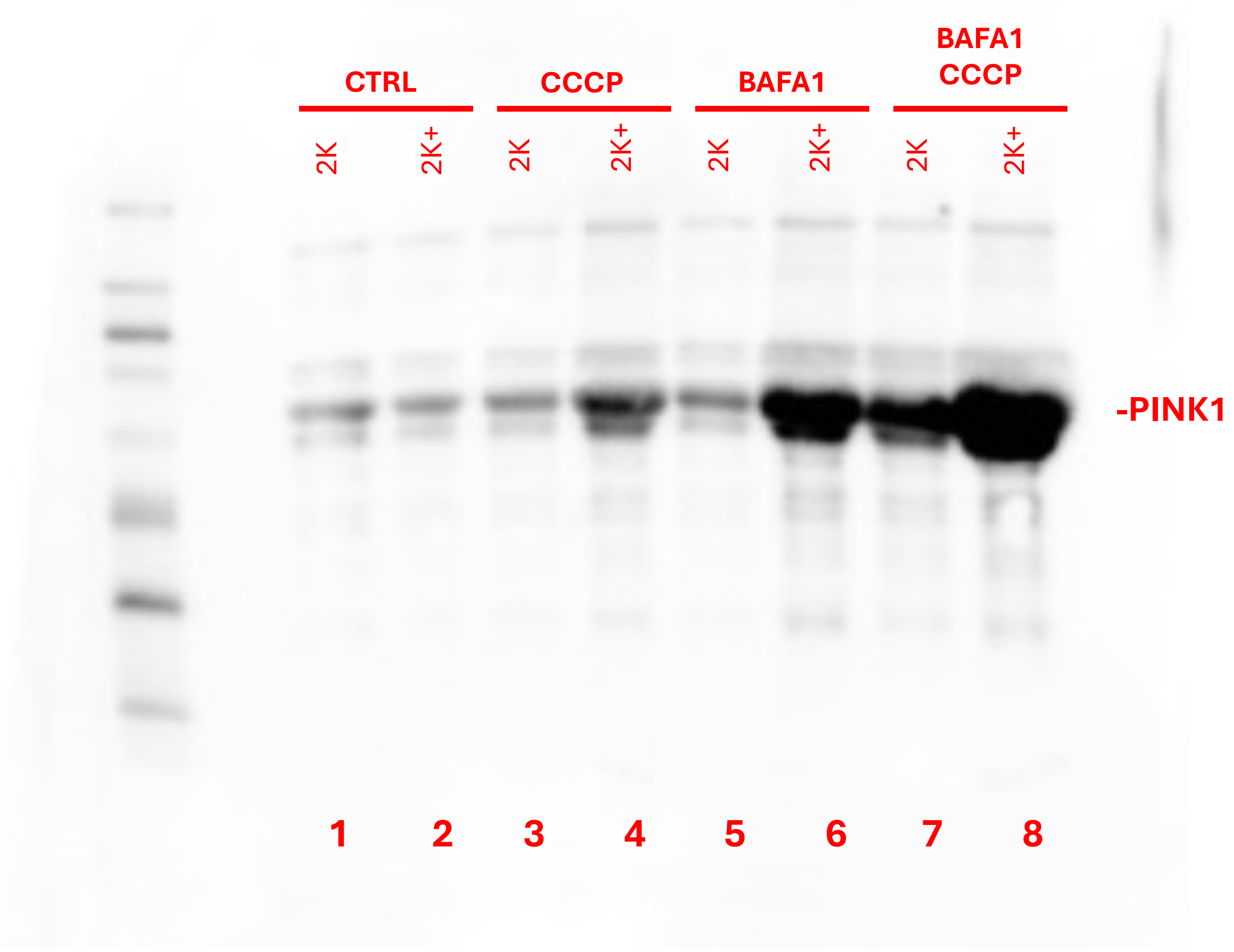

Full western blot image presented in Figure 3B

4T1 treatment PINK1

(Trial 3)

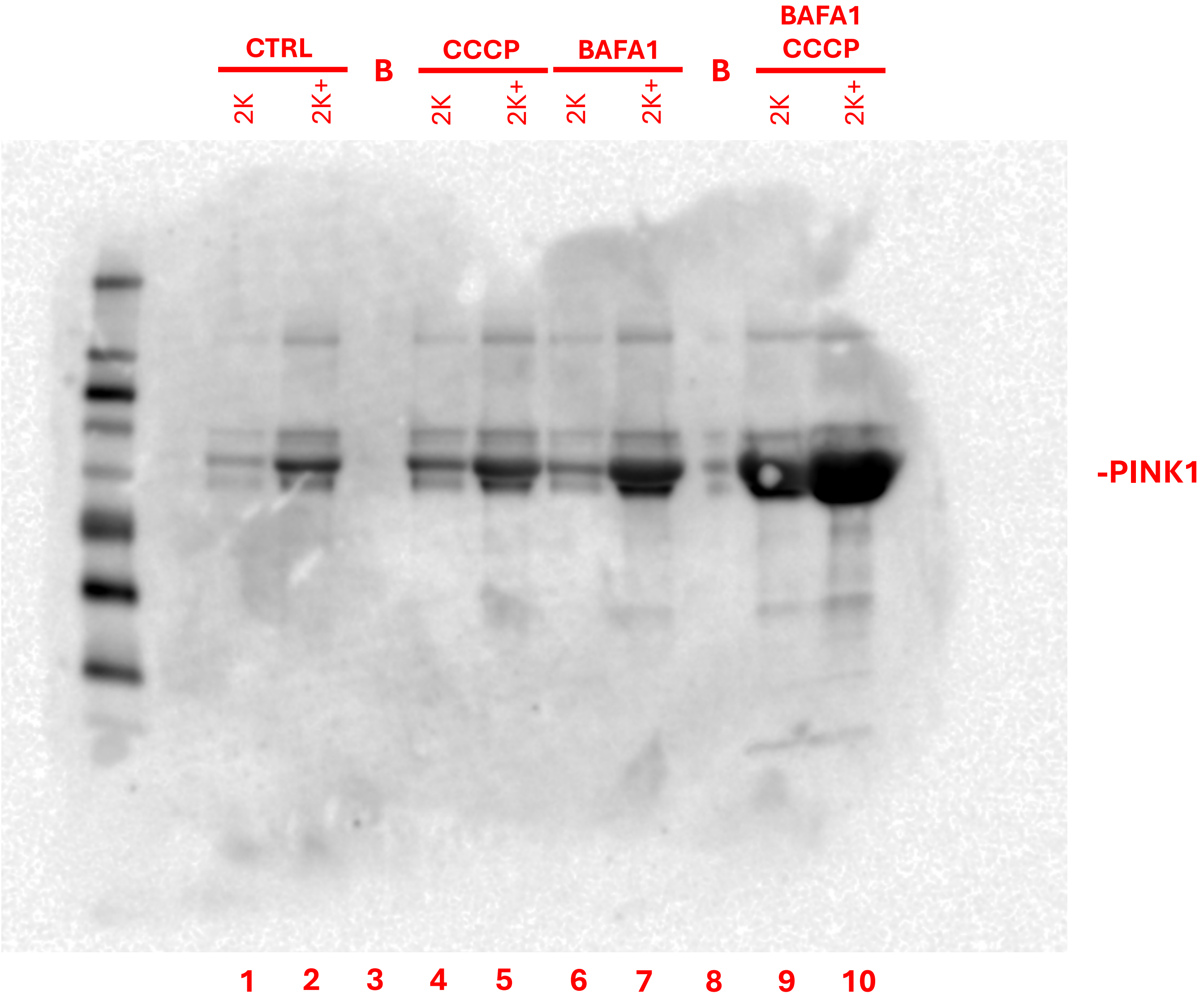

Biological replicate 2 (PINK1) for western blot presented in Figure 3B

4T1 treatment CD81

(Trial 3)

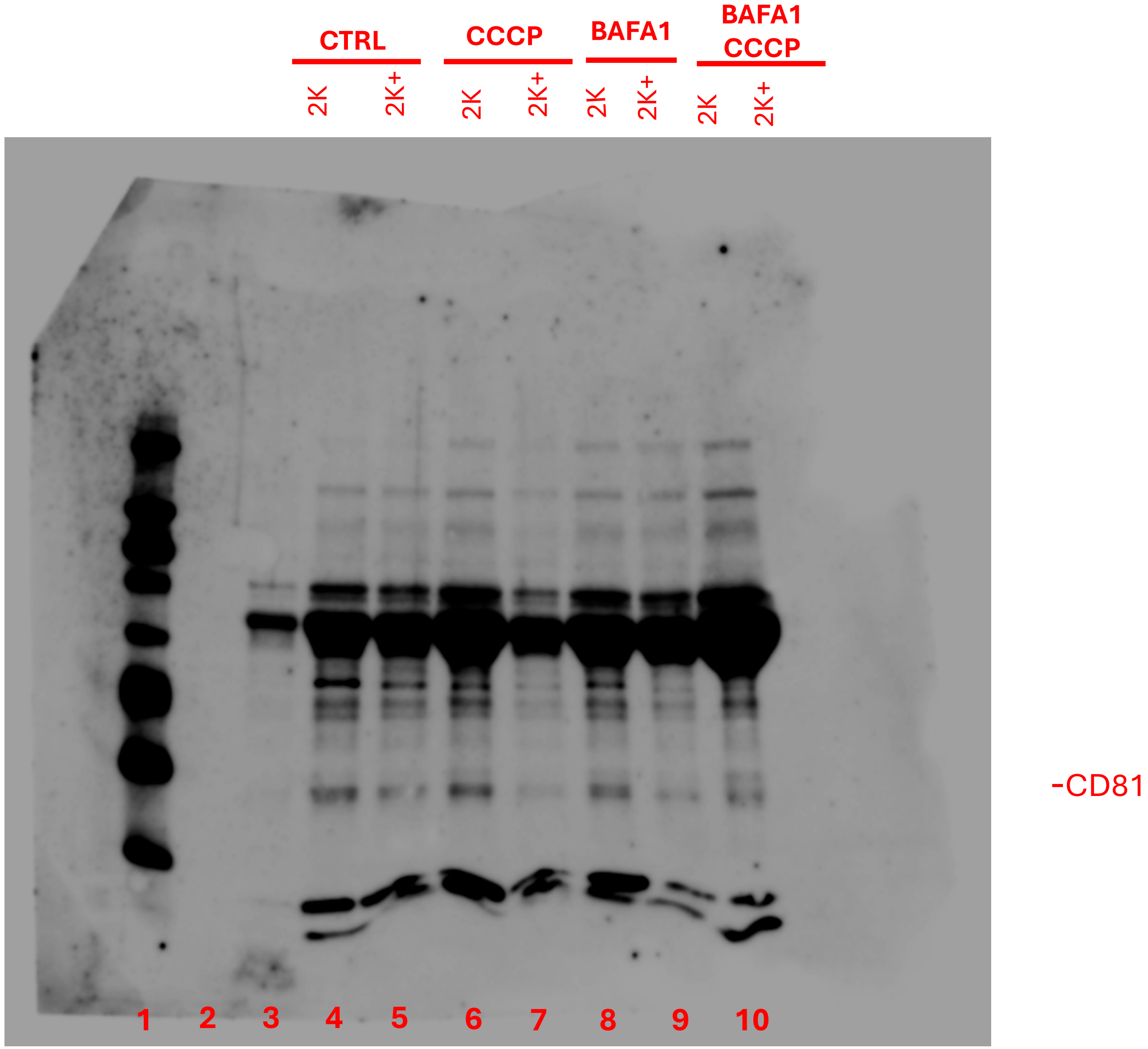

Full western blot image presented in Figure 3B

4T1 treatment CD81

(Trial 3)

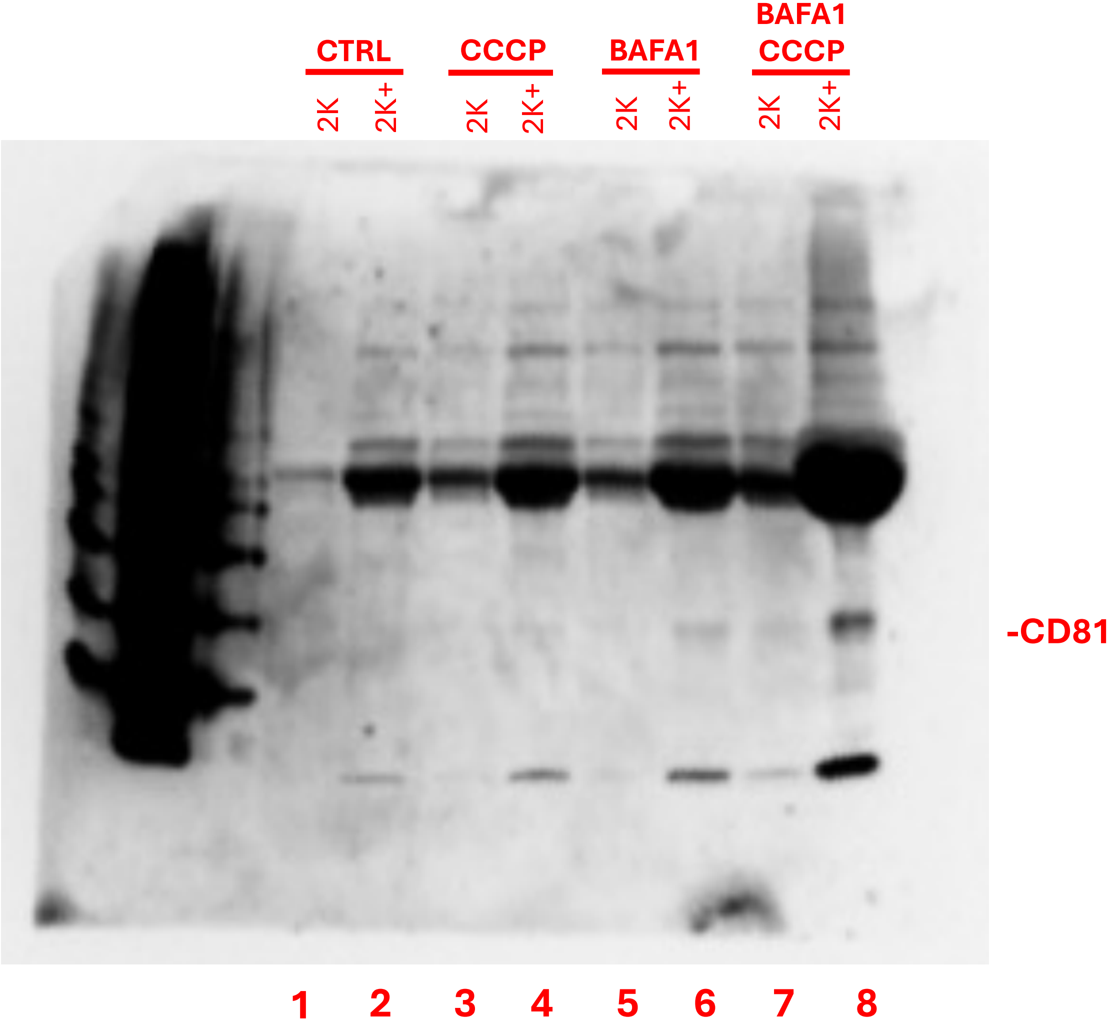

Biological replicate 1 (CD81) for western blot presented in Figure 3B

4T1 treatment Actin

(Trial 2)

Stripped from
PINK1 trial 2

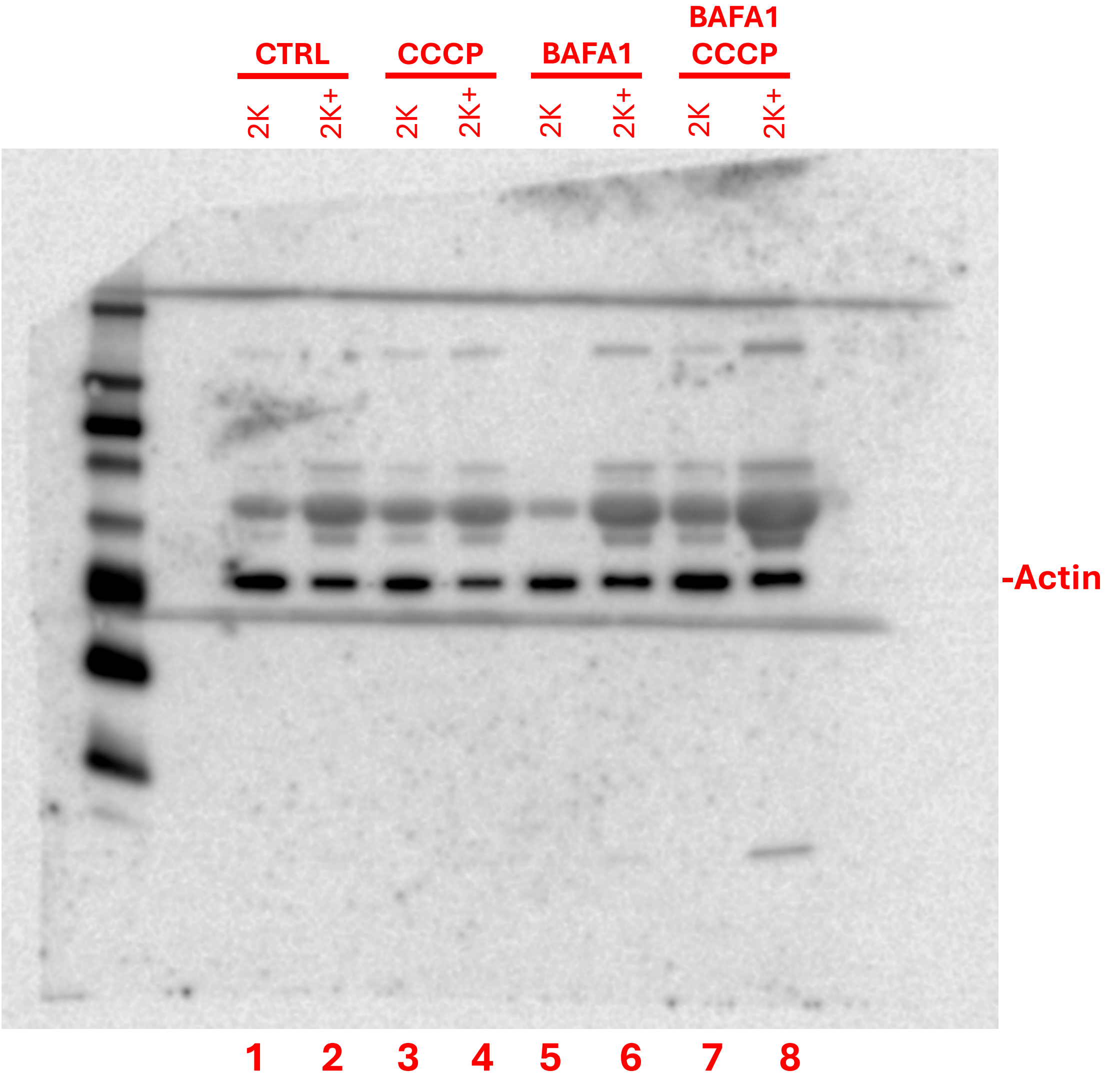

Full western blot image presented in Figure 3B

4T1 treatment Actin

(Trial 3)

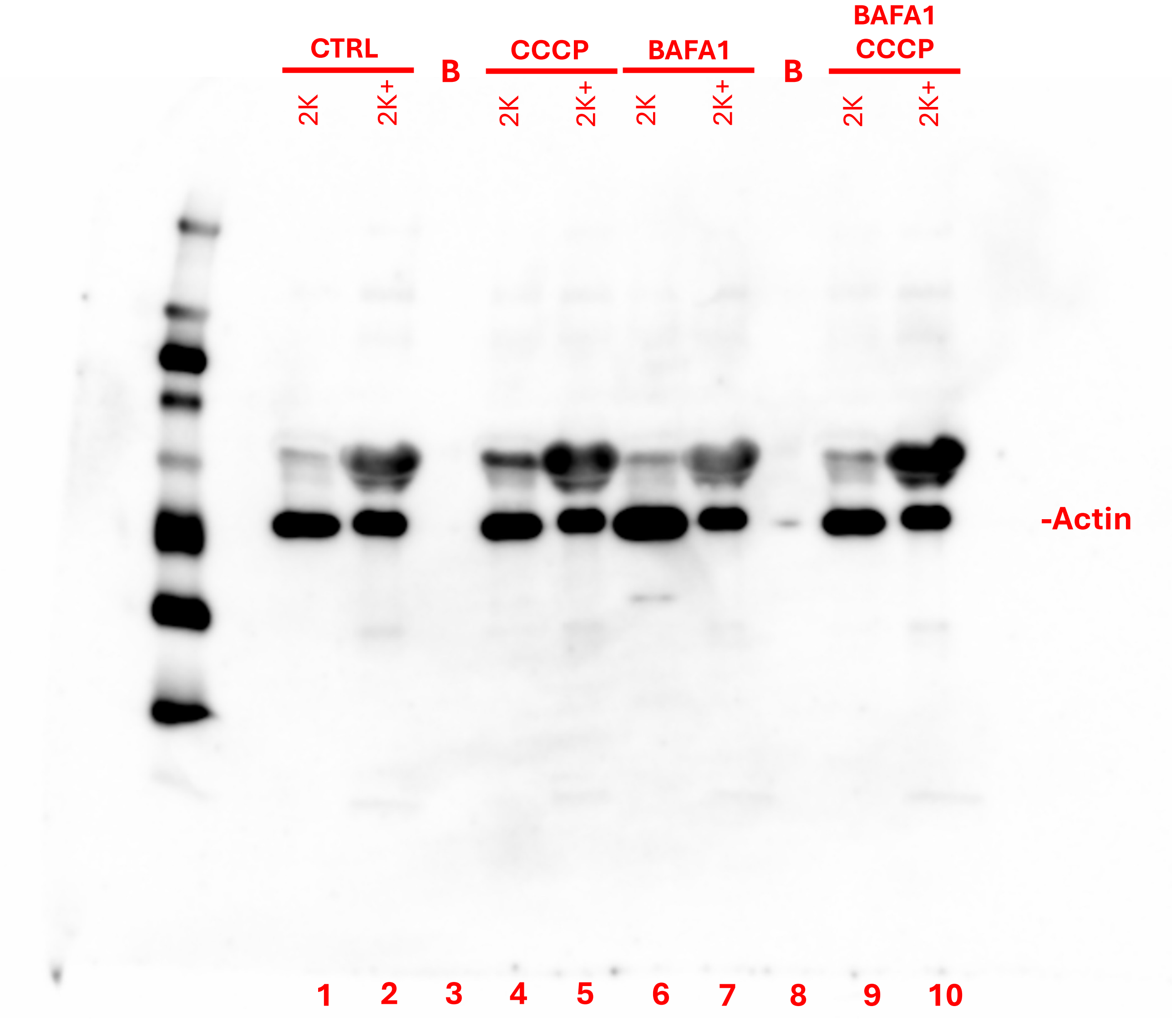

Biological replicate 1 (Actin) for western blot presented in Figure 3B

IOMM-lee dose response- PINK1

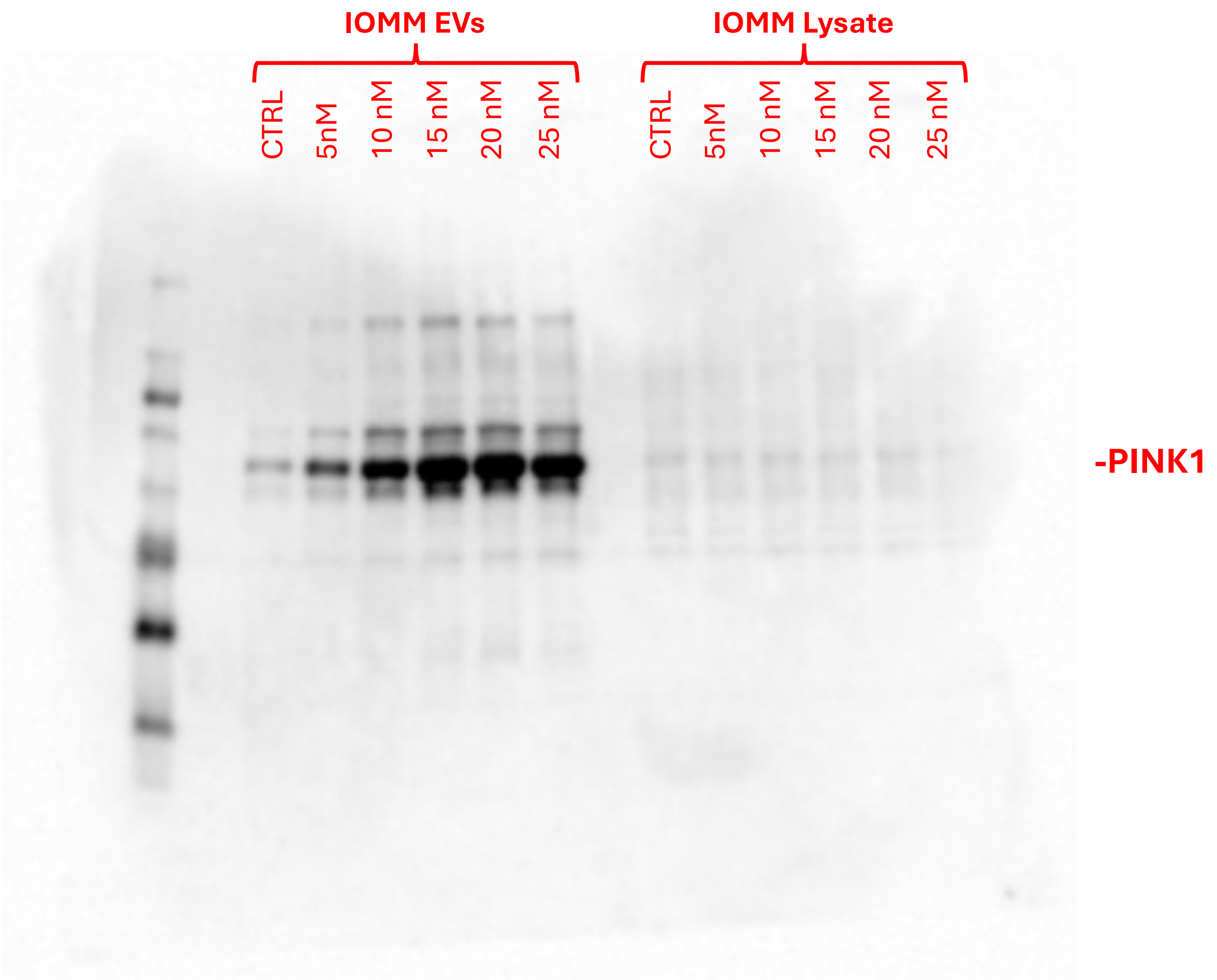

Full western blot image presented in Figure 3E

IOMM-lee dose response- CD81

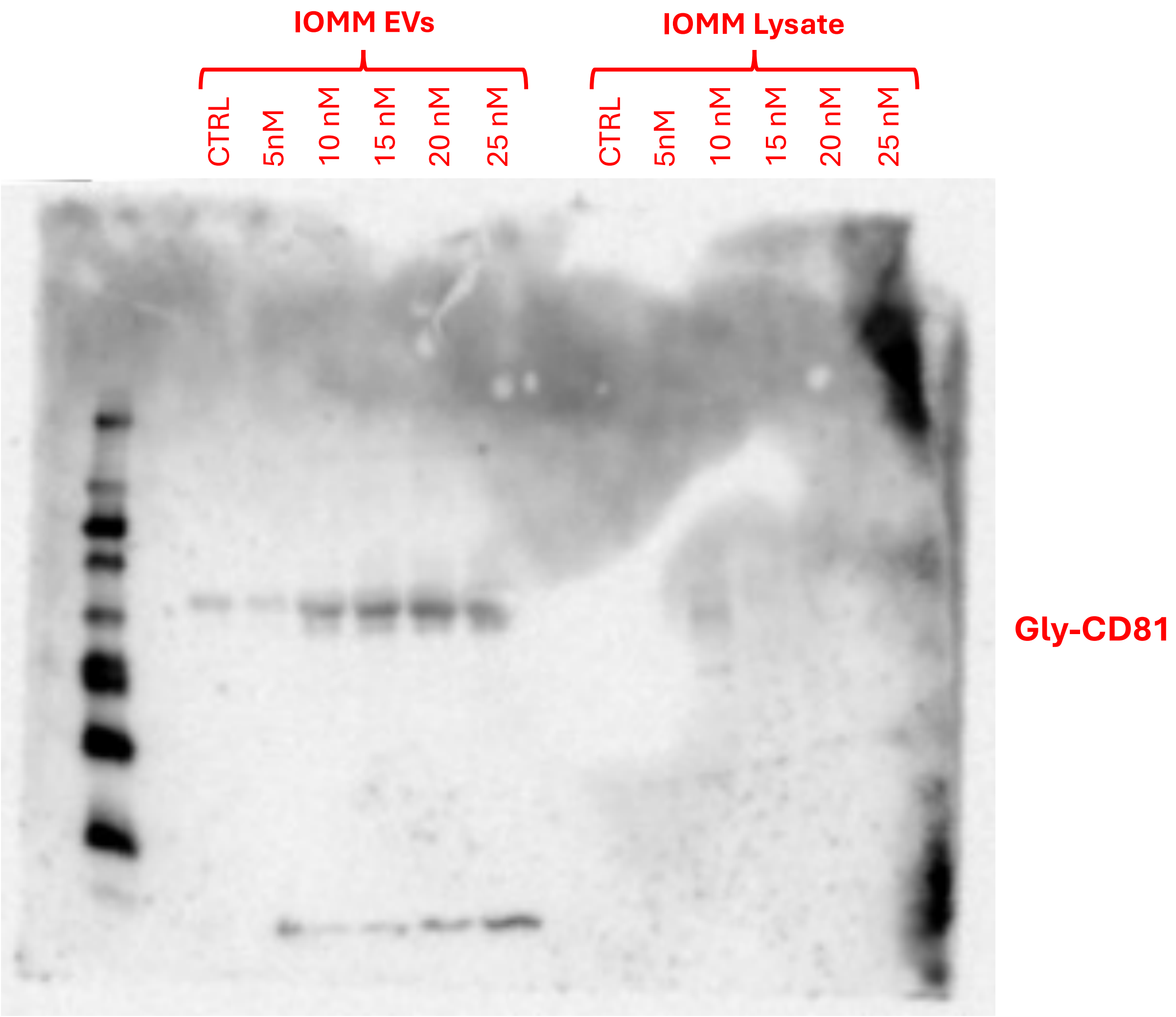

Full western blot image presented in Figure 3E

IOMM-lee dose response- Calnexin

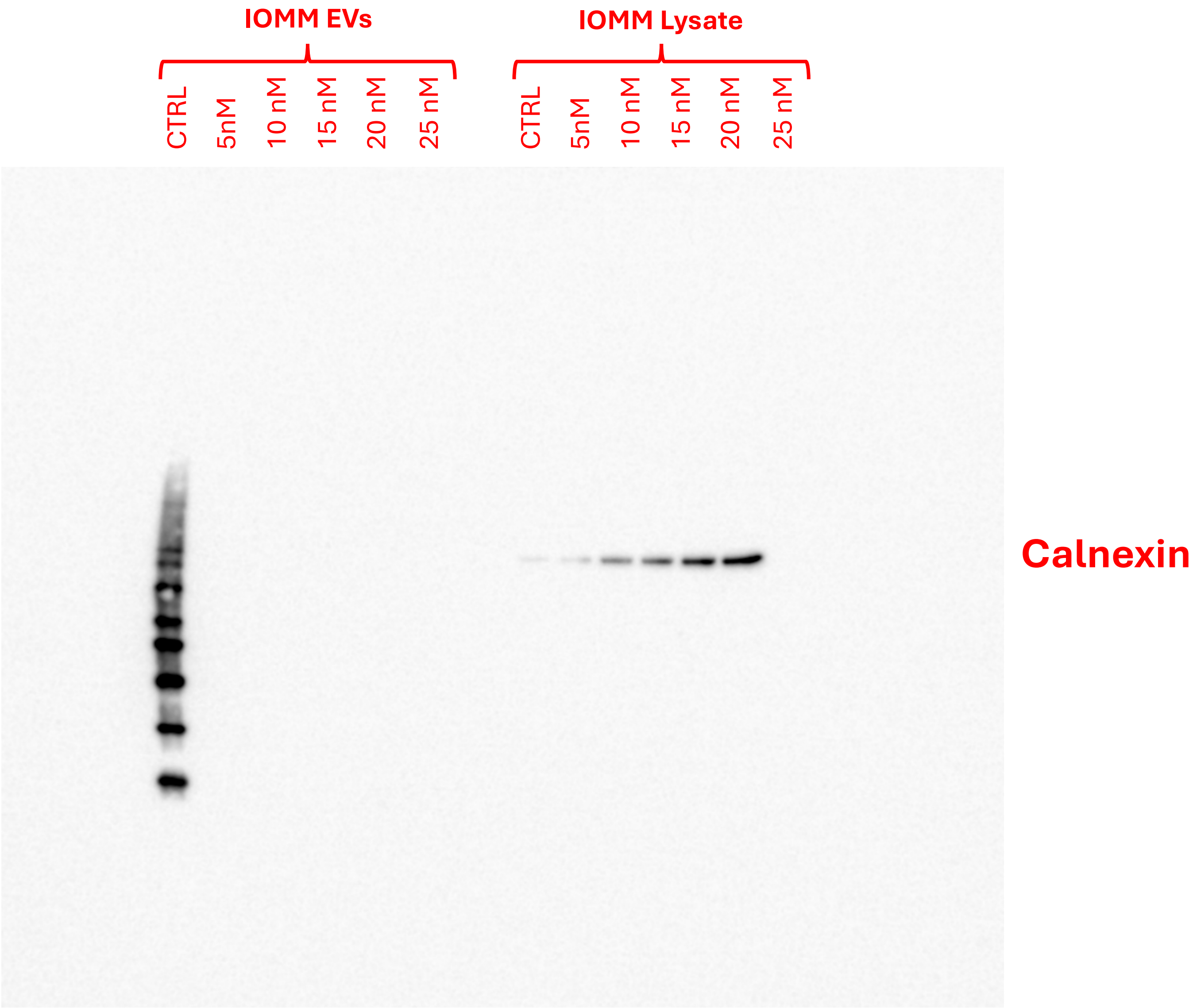

Full western blot image presented in Figure 3E

IOMM-lee dose response- Actin

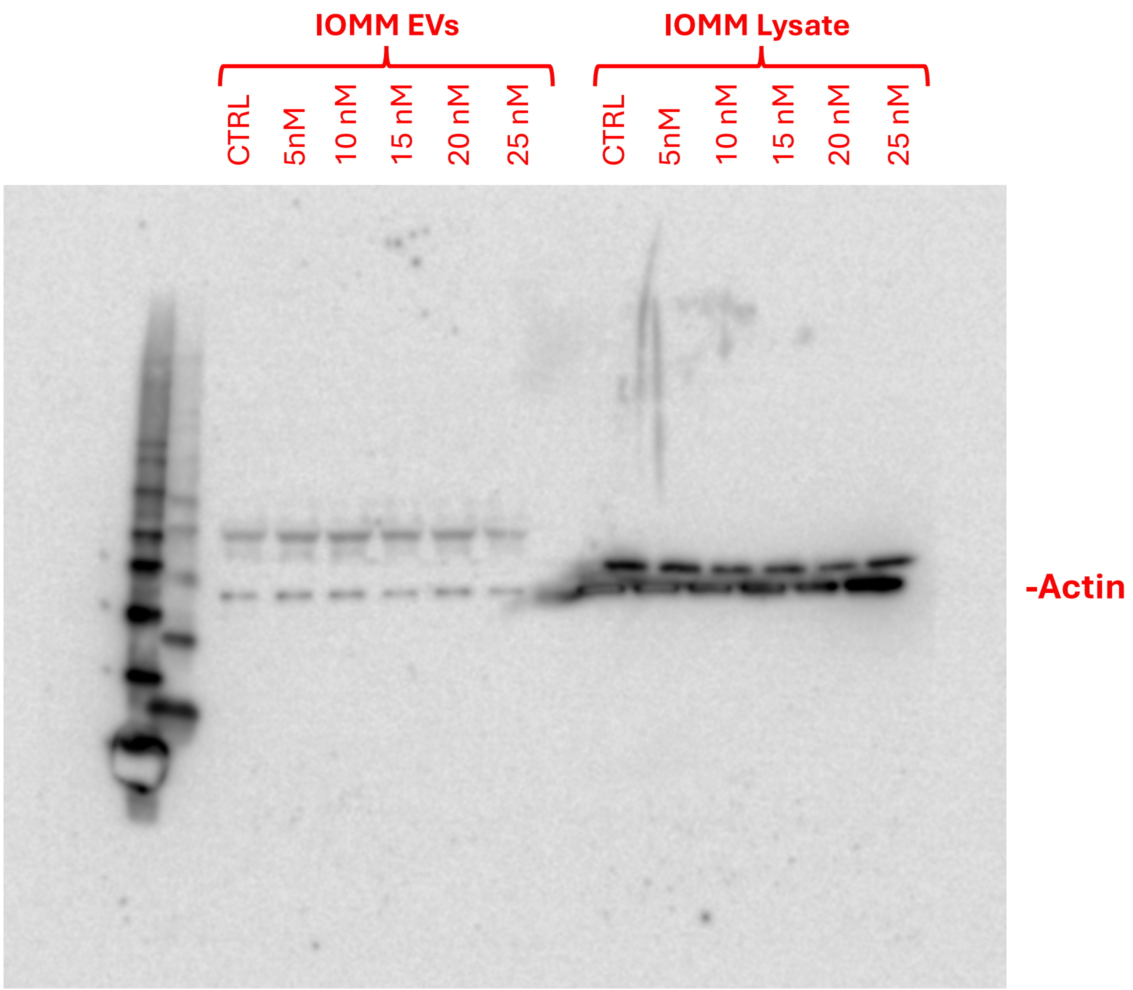

Full western blot image presented in Figure 3E

IOMM Treatment PINK1

(Trial 2)

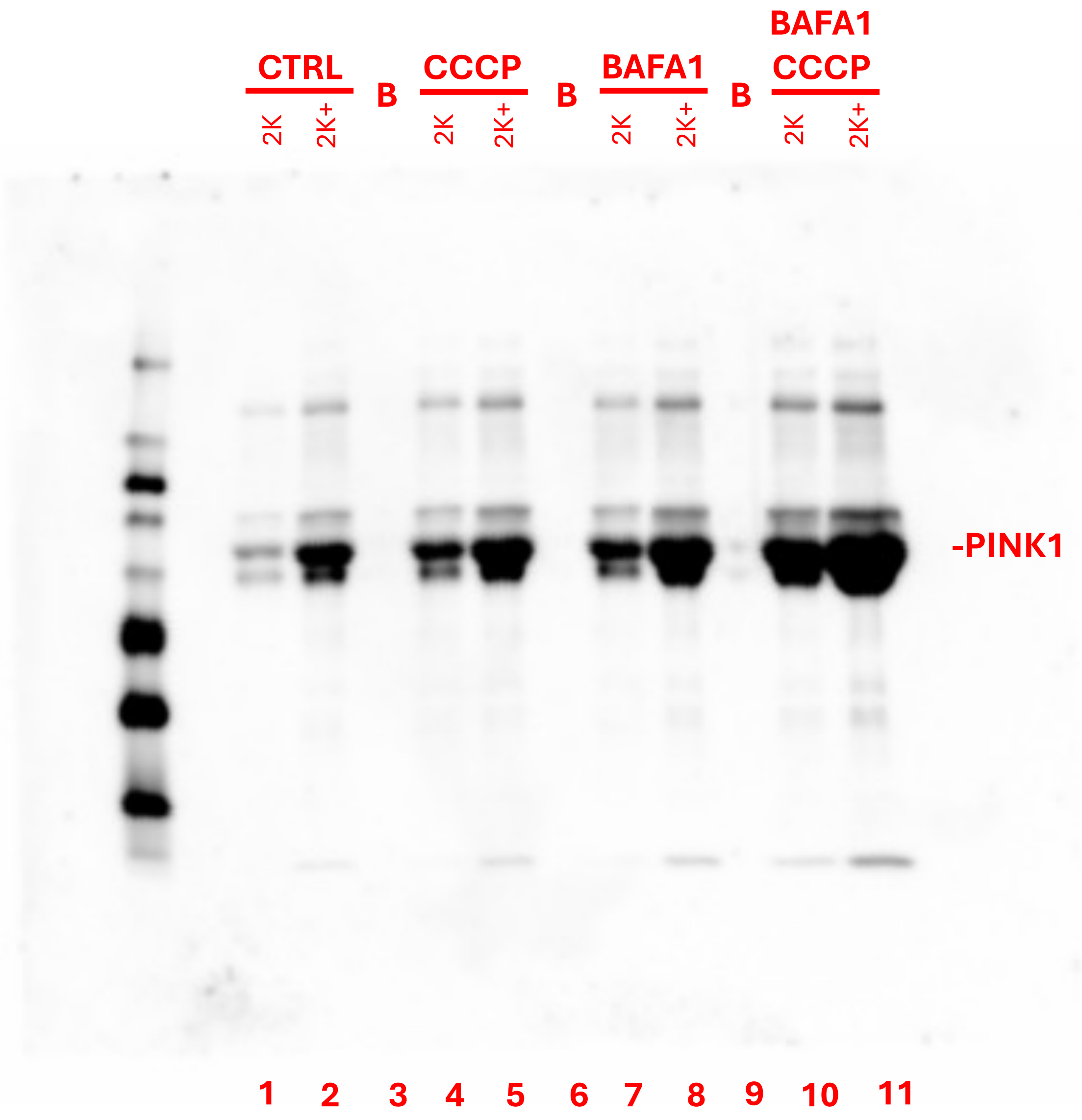

Full western blot image presented in Figure 3F

IOMM Treatment PINK1

(Trial 1)

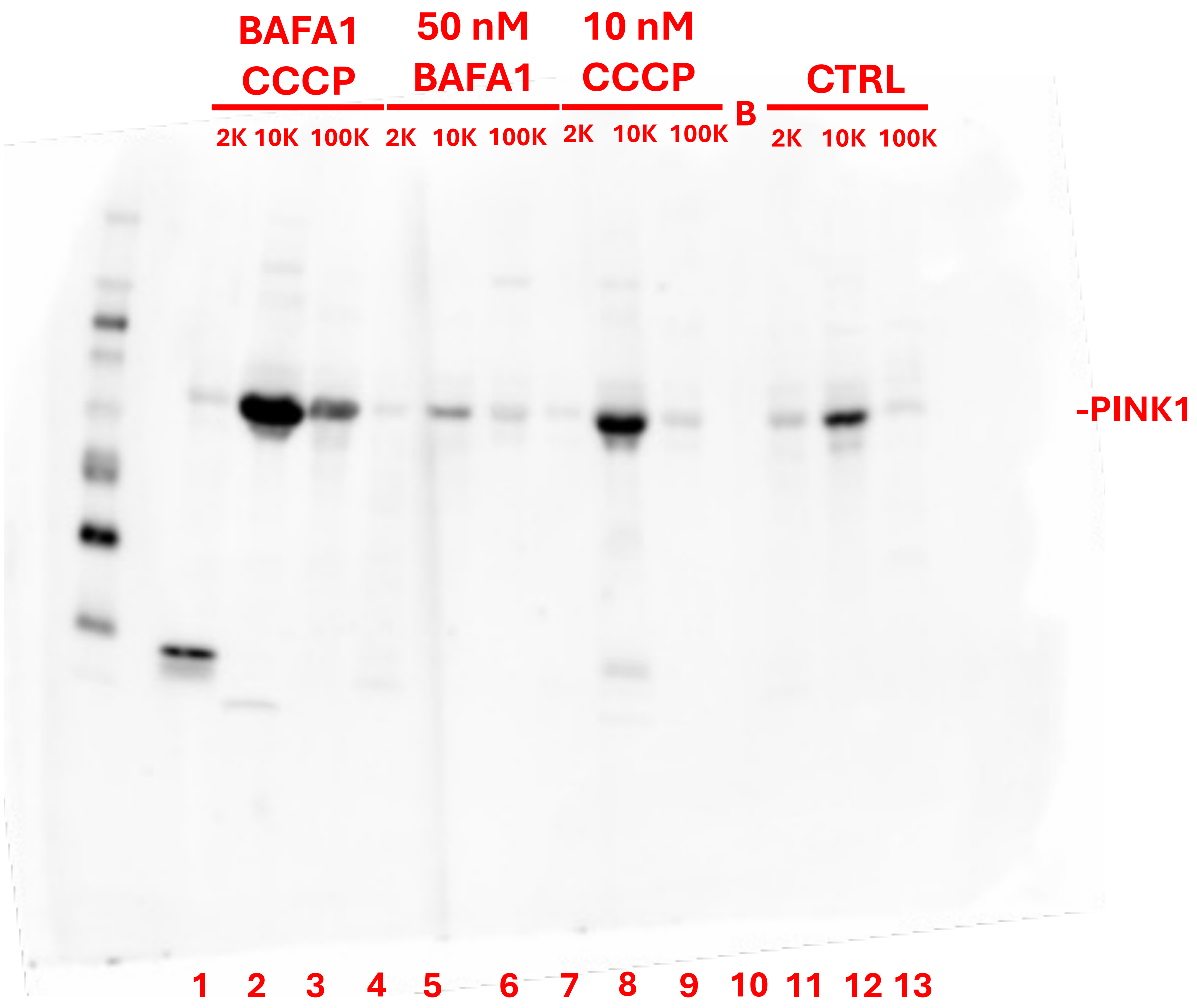

Biological replicate 1 (PINK1 for western blot presented in Figure 3F

IOMM Treatment CD81

(Trial 2)

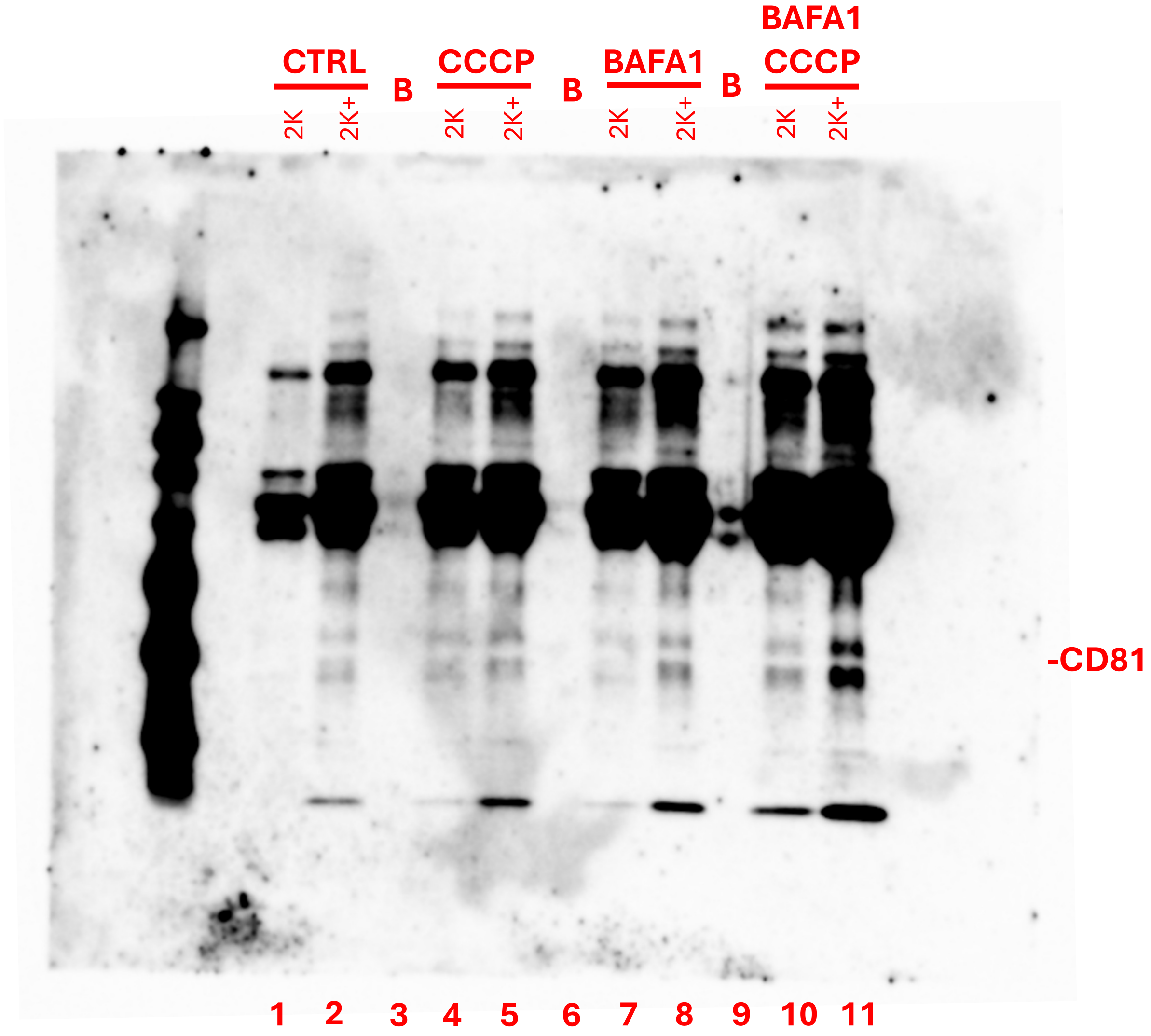

Full western blot image presented in Figure 3F

IOMM Treatment CD81

(Trial 1)

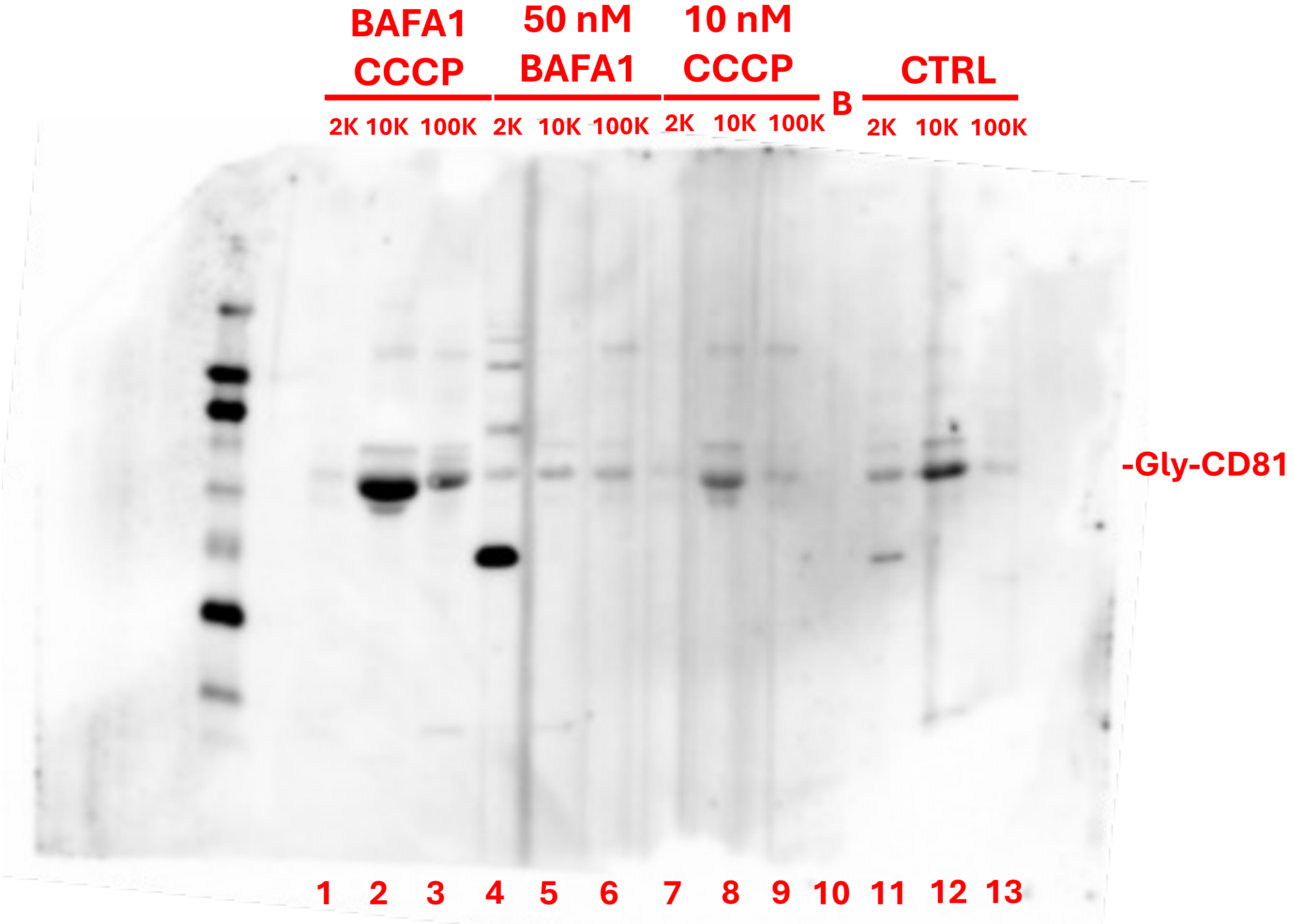

Biological replicate 1 (CD81) for western blot presented in Figure 3F

IOMM Treatment Actin

(Trial 2)

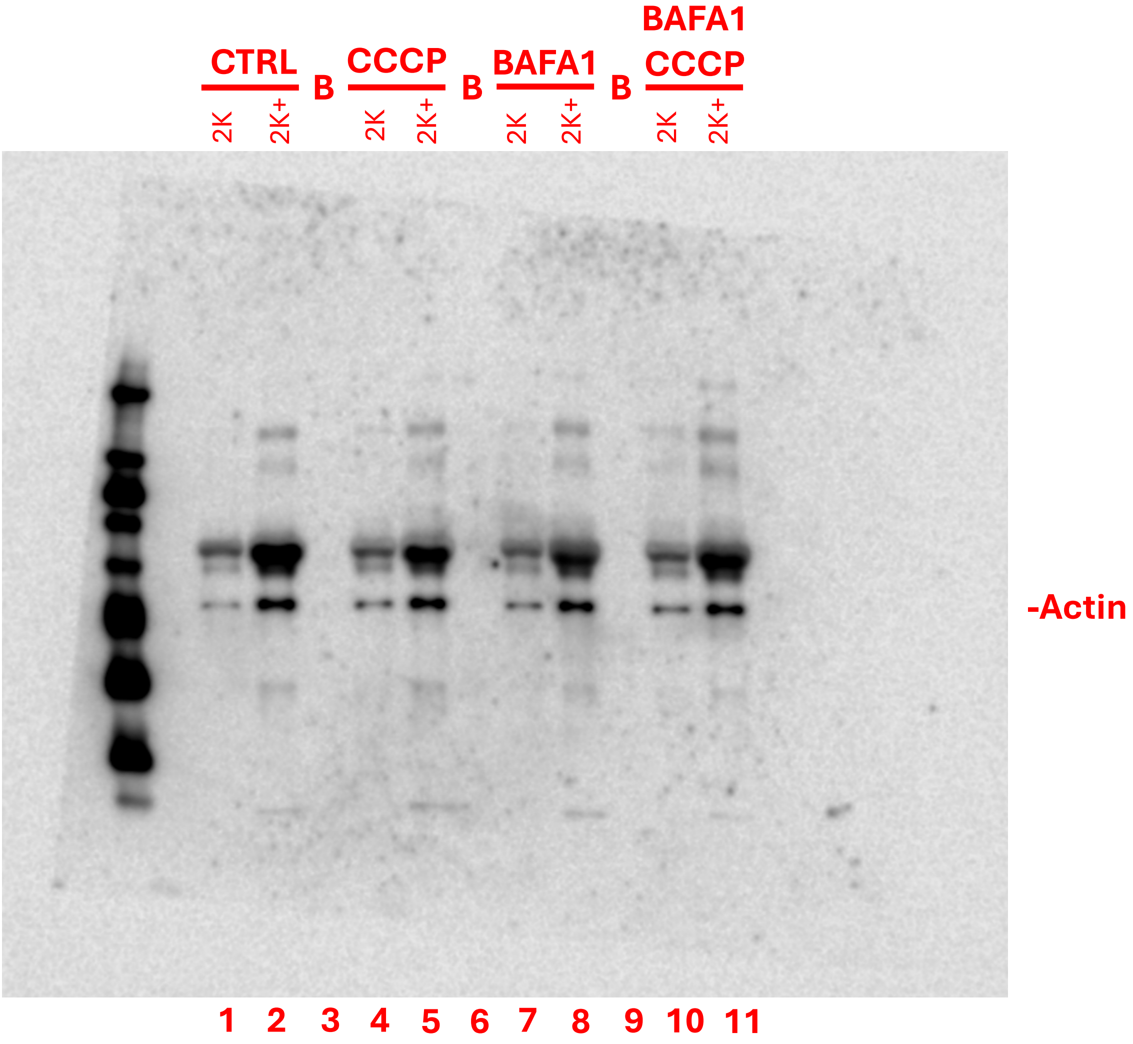

Full western blot image presented in Figure 3F

IOMM Treatment Actin

(Trial 1)

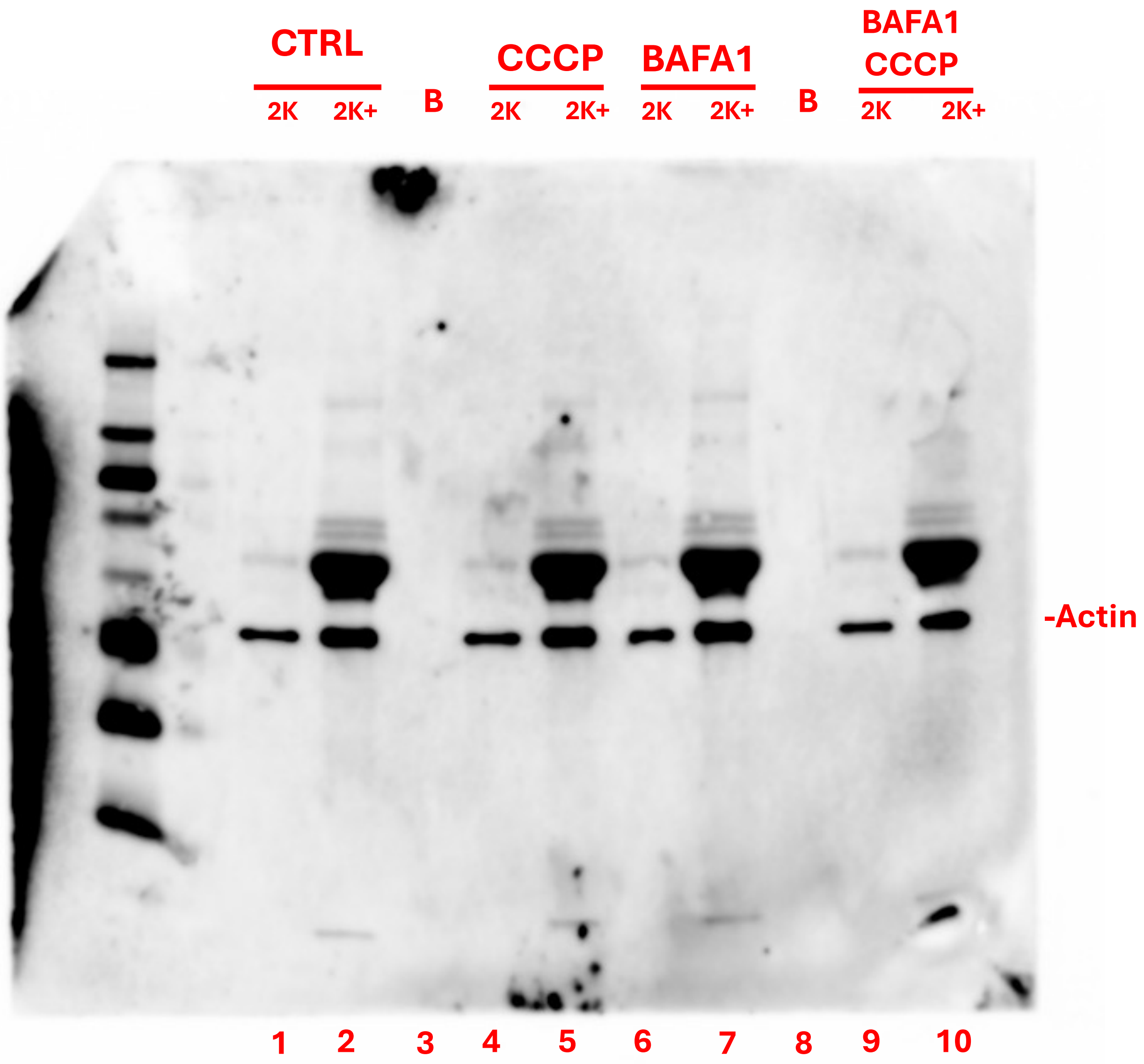

Biological replicate 1 (Actin) for western blot presented in Figure 3F

CCCP and Valinomycin blots – Supple. Figure 1

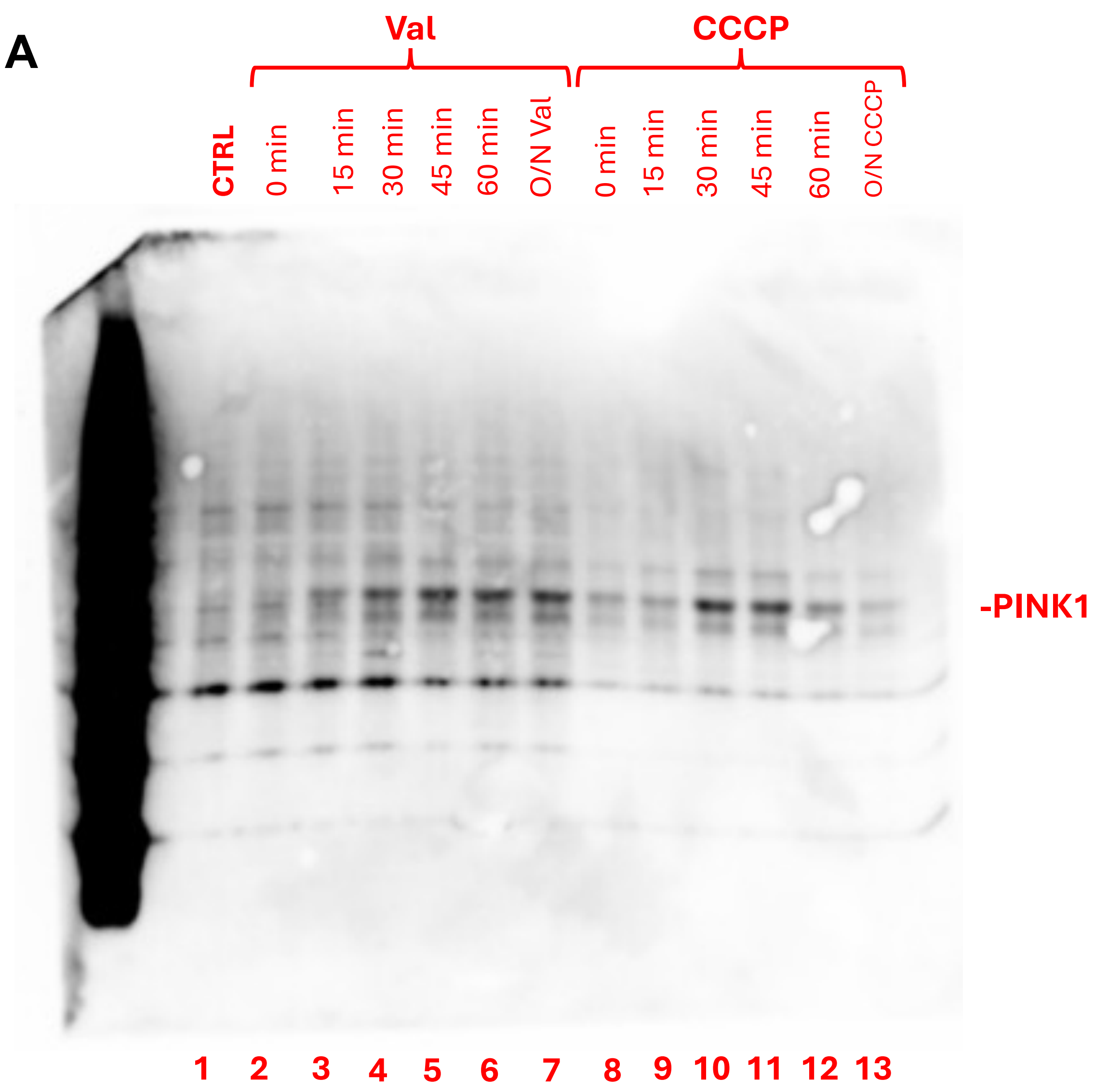

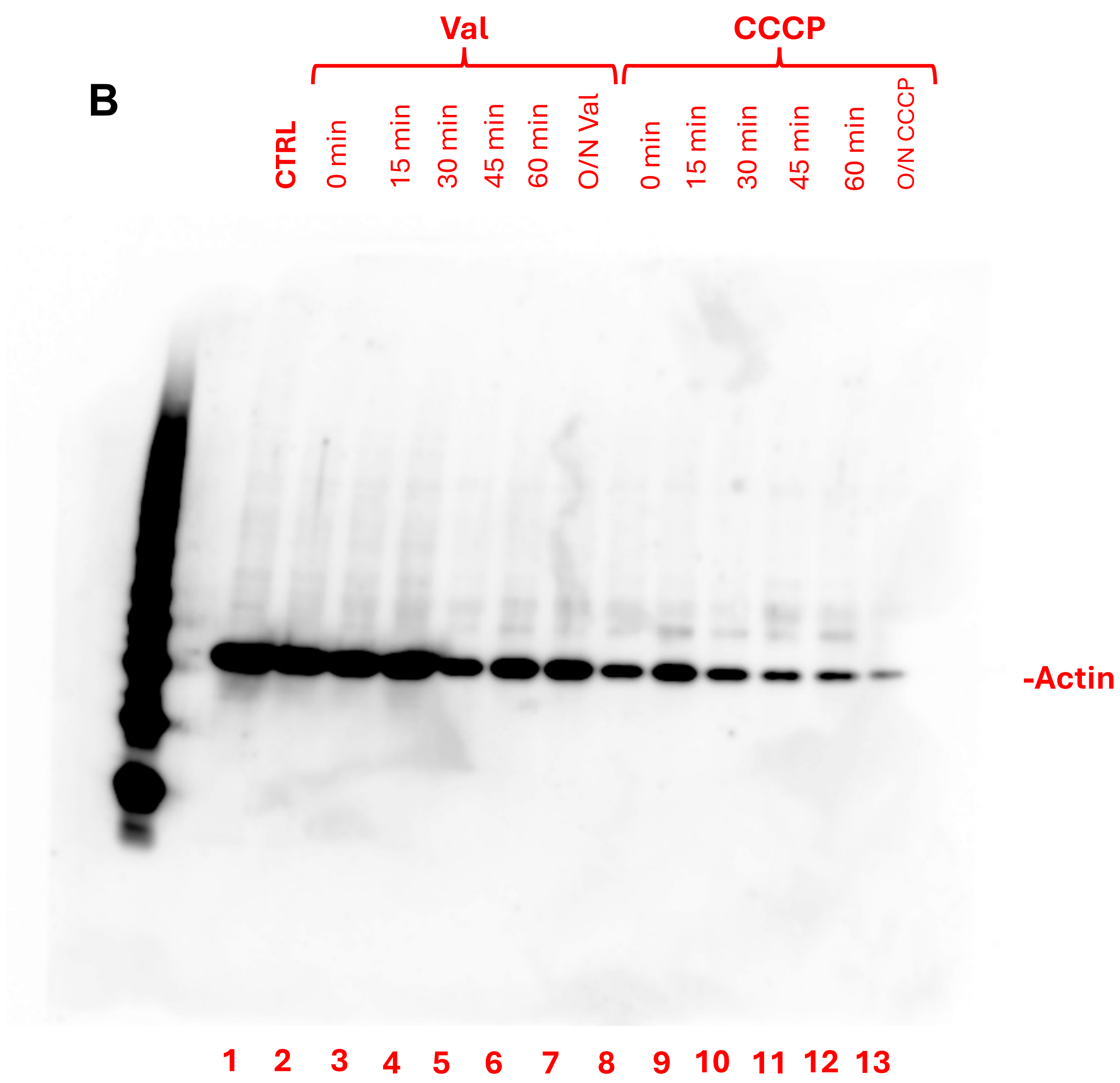

Supplement: Supplementary file 3 [file DataSheet1.pdf]
